# Supplementary material for: Molecular Signatures of Regression of the Canine Transmissible Venereal Tumor
Source: Cancer Cell. 2018 Apr 9;33(4):620–633.e6. doi: 10.1016/j.ccell.2018.03.003 (PMC5896242; doi:10.1016/j.ccell.2018.03.003)
Supplement: Document S1. Figures S1–S5 [file mmc1.pdf]

Cancer Cell, Volume *33*

Supplemental Information

Molecular Signatures of Regression of the Canine Transmissible Venereal Tumor

Dan Frampton, Hagen Schwenzer, Gabriele Marino, Lee M. Butcher, Gabriele Pollara, Janos Kriston-Vizi, Cristina Venturini, Rachel Austin, Karina Ferreira de Castro, Robin Ketteler, Benjamin Chain, Richard A. Goldstein, Robin A. Weiss, Stephan Beck, and Ariberto Fassati

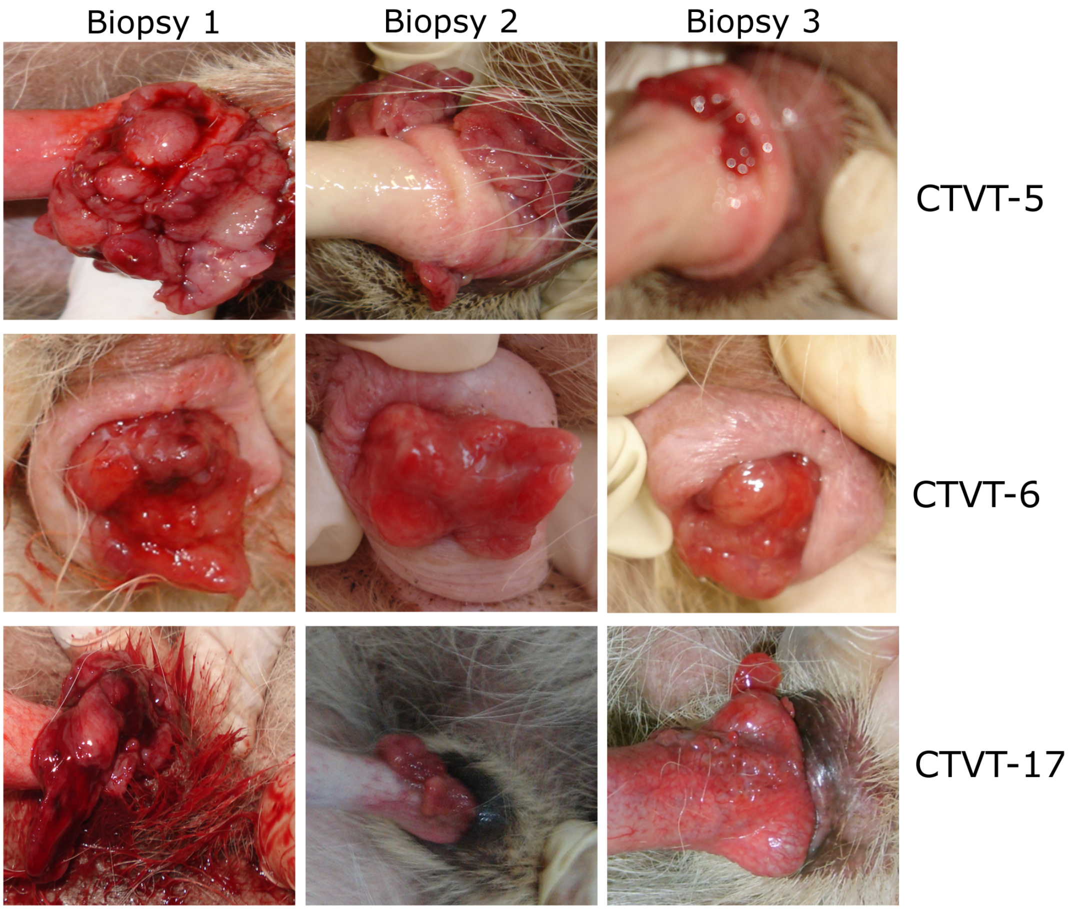

Figure S1 (related to Table 1).

Macroscopical appearance of CTVT cases diagnosed in Italy and used in this study for RNAseq and MeDIP-seq. CTVT-5 and CTVT-17 were diagnosed in male mixed breed dogs and CTVT-6 in a female mixed breed dog. Biopsies were collected at day 0, day 6 and day 14 after vincristine administration (CTVT-5 and CTVT-6), or at day 0, 22 and 48 after vincristine administration (CTVT-17). At the latest time point, CTVT-5 and CTVT-17 show overt clinical regression whereas CTVT-6 has clinical appearance of a tumor in stationary phase.

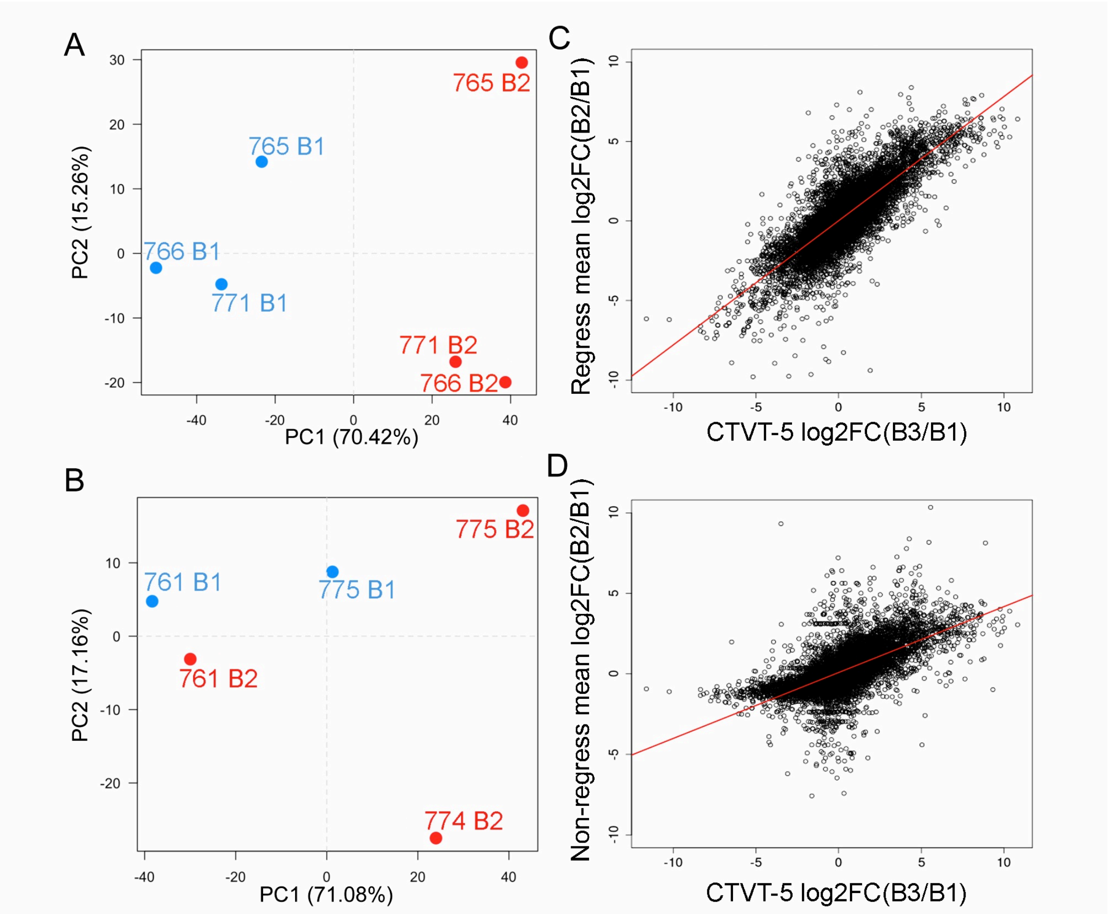

Figure S2 (related to Figure 2). Correlative analysis of regressive and non-regressive CTVTs.

(A, B) Principal component analysis (PCA) for the 7xx regressive (A) and non-regressive CTVTs (B). The figure shows the first two components of PCA with the percentage of variance associated with each axis. The first principal component (PC1) is expected to separate samples based on the different biological conditions, in blue biopsy 1 (B1) and in red biopsy 2 (B2). (C) correlation between the log2 fold-changes in gene expression in biopsy 3 (B3) relative to biopsy 1 (B1) of CTVT-5 (x-axis) and the mean log2 fold-changes in B2 relative to B1 in the regressive 7xx CTVTs. (D) correlation between the log2 fold-changes in gene expression in B3 relative to B1 of CTVT-5 (x-axis) and the mean log2 fold-changes for B2 relative to B1 in the non-regressive 7xx CTVTs. Pearson's correlation coefficient was calculated for both comparisons. Each dot represents a gene.

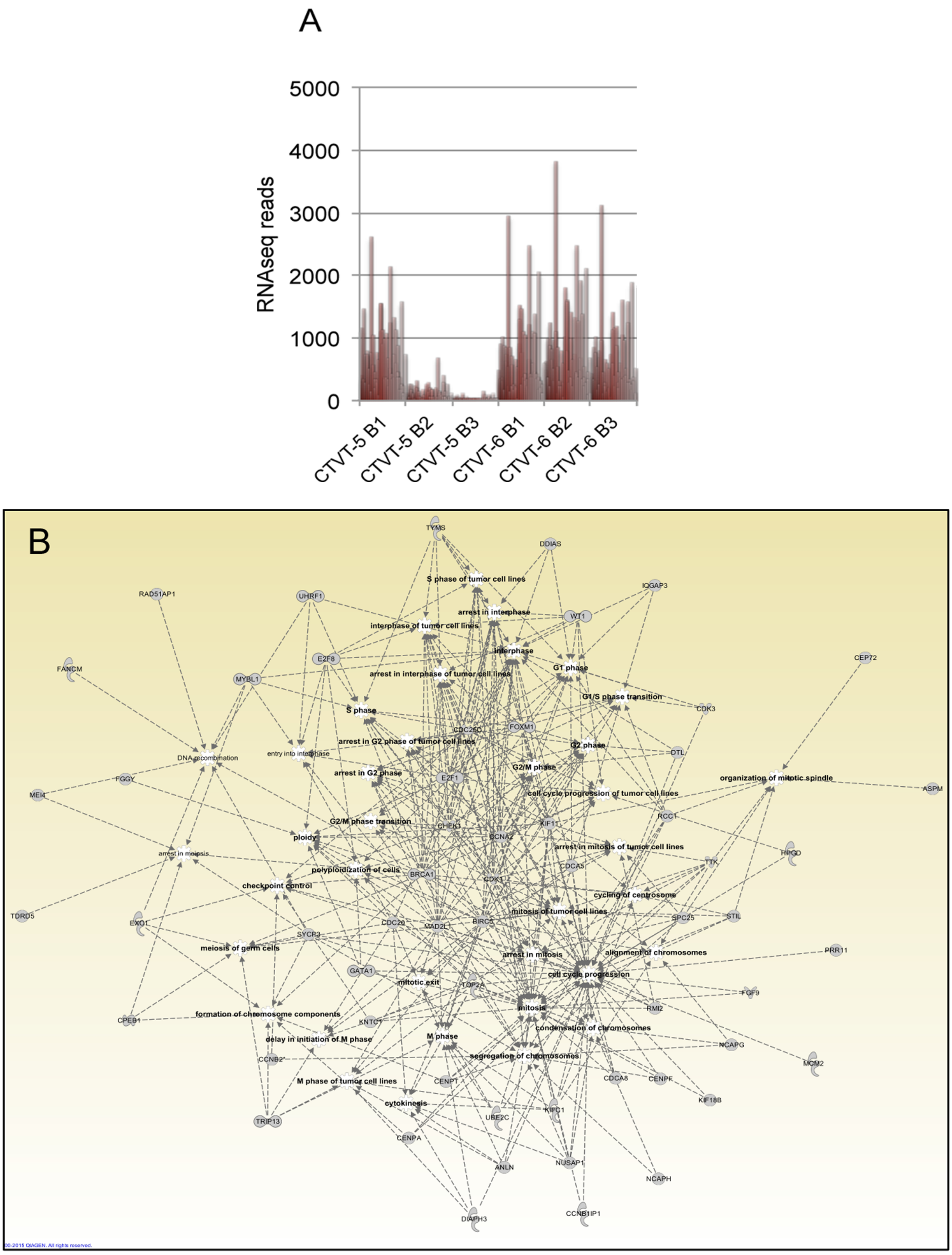

Figure S3 (related to Figure 6). Downregulation of cell cycle-related genes.

(A) Plot showing down-regulation of 60 cell cycle-related genes across the sequential biopsies of CTVT-5 and CTVT-6. B1: biopsy 1; B2: biopsy 2; B3: biopsy 3. The full list of

genes in provided in Table S7. (B) IPA diagram showing cell cycle gene networks of progressive downregulated genes in CTVT-5.

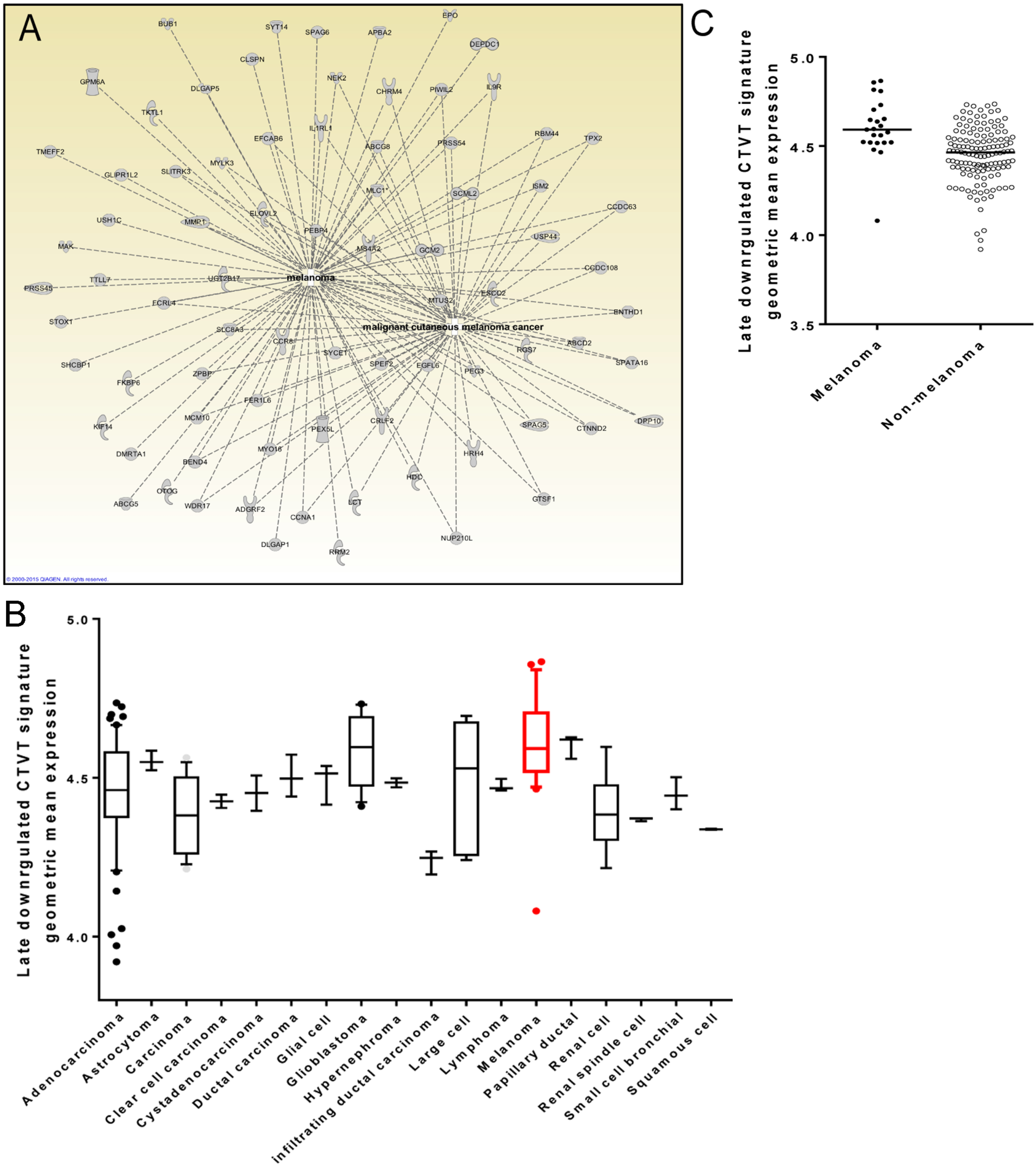

Figure S4 (related to Figure 6). Late down-regulated genes show a melanoma-like signature.

(A) IPA diagram showing melanoma/skin cancer gene networks of late downregulated genes in the third CTVT-5 biopsy. (B) Expression of late downregulated CTVT signature in NCI-60 cancer cell lines. Geometric mean expression of signature in multiple solid organ

cancer cell lines. Box represents the interquartile range and line represents median value. Whiskers are drawn to the 10th & 90th percentiles. (C) Comparison in geometric mean expression of late downregulated CTVT signature in melanoma cancer cells relative to all other solid organ cancer cells. Horizontal line represents median values, $p < 0.0001$ by Mann-Whitney test.

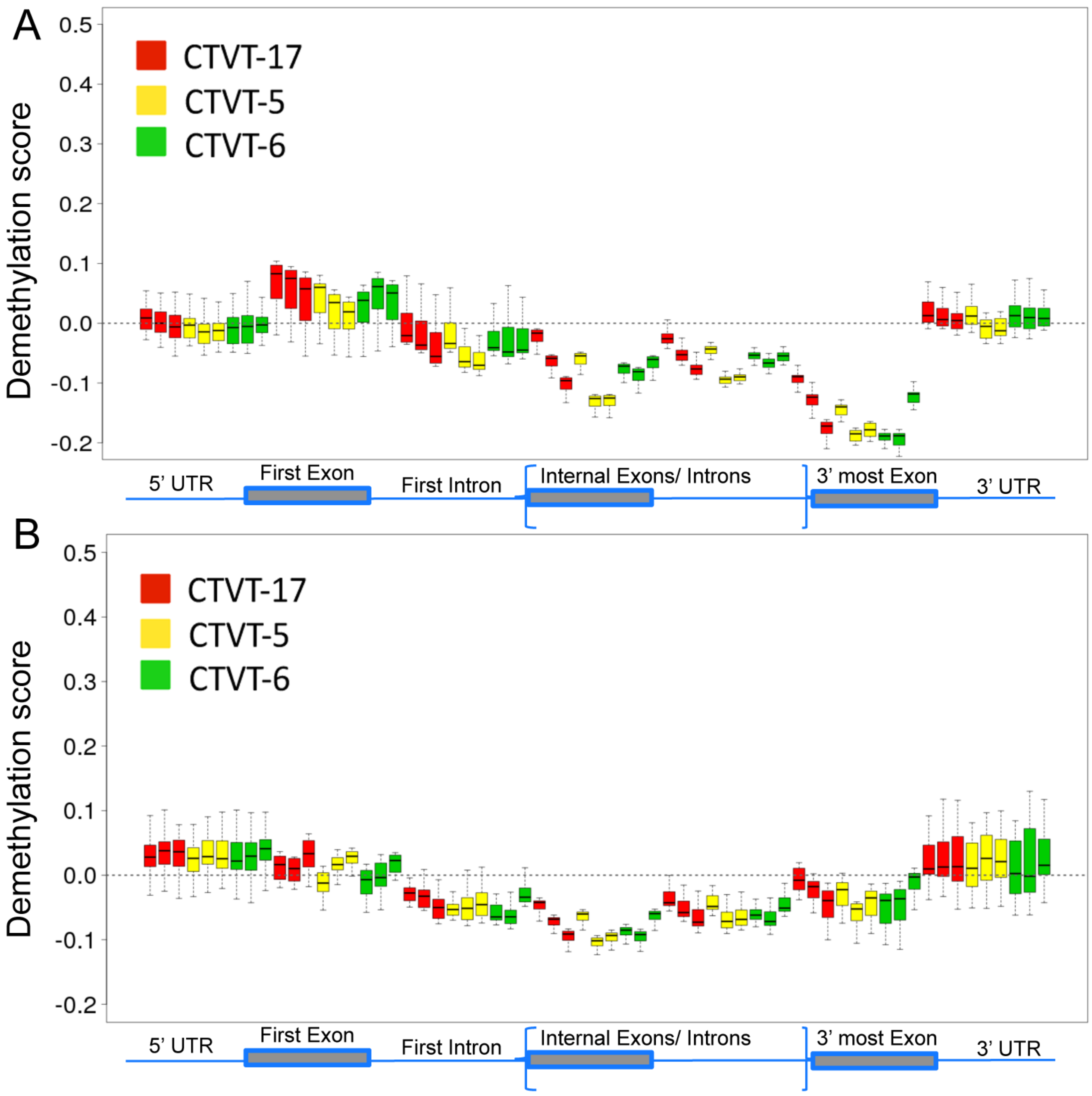

Figure S5 (related to Figure 7). Changes in gene expression correlate with specific changes in DNA methylation.

(A-B) De-methylation profiles of “early” (A) and “late” (B) downregulated genes across serial biopsies of CTVT-17 (red), CTVT-5 (yellow) and CTVT-6 (green). De-methylation scores were obtained for individual genes by quantifying demethylation levels within specific regions of genes (≤2 kb upstream of first exon; first exon; first intron; internal exons; internal introns; last exon; ≤2 kb downstream of last exon) and normalized by subtracting the corresponding demethylation values observed for non-expressed genes. Boxplots illustrate the variation within these values across each gene-list (boxes extend to the first and third

quartile, whiskers extend to 1.5x inter-quartile range and line represents median values). For each CTVT sample, boxplots are in order (from left to right): 1st, 2nd and 3rd biopsy.
