# Supplementary material for: Molecular Signatures of Regression of the Canine Transmissible Venereal Tumor
Source: Cancer Cell. 2018 Apr 9;33(4):620–633.e6. doi: 10.1016/j.ccell.2018.03.003 (PMC5896242; doi:10.1016/j.ccell.2018.03.003)
Supplement: Document S2. Article plus Supplemental Information [file mmc10.pdf]

Cancer Cell

Article

Molecular Signatures of Regression of the Canine Transmissible Venereal Tumor

Graphical Abstract

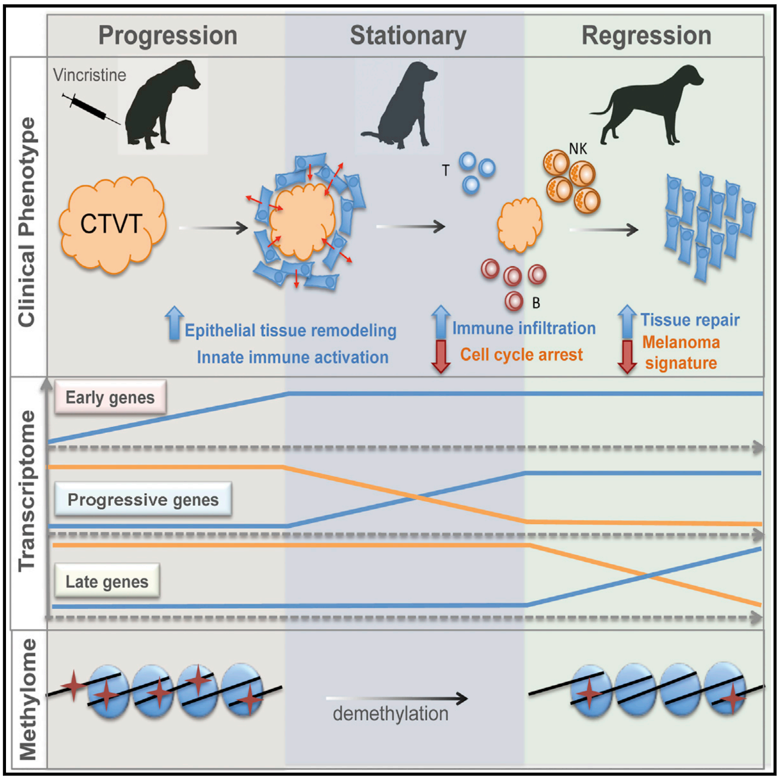

Highlights

- The canine transmissible venereal tumor regresses after chemotherapy
- Regression correlates with early upregulation of inflammation genes by host
- Host cells surrounding tumor upregulate specific chemokine genes
- Chemokines trigger invasion of CD8, CD4, NK, and B cells and tumor clearance

Authors

Dan Frampton, Hagen Schwenzer, Gabriele Marino, ..., Robin A. Weiss, Stephan Beck, Ariberto Fassati

Correspondence

a.fassati@ucl.ac.uk

In Brief

By analyzing serial biopsies of vincristine-treated canine transmissible venereal tumors, Frampton et al. show that tumor regression occurs in sequential steps involving the activation of the innate immune system and immune infiltration of the tumor, and they identify CCL5 as a possible driver of regression.

Frampton et al., 2018, Cancer Cell *33*, 620–633
April 9, 2018 © 2018 The Author(s). Published by Elsevier Inc.
https://doi.org/10.1016/j.ccell.2018.03.003

Cancer Cell
Article

Molecular Signatures of Regression of the Canine Transmissible Venereal Tumor

Dan Frampton,[1] Hagen Schwenzer,[1] Gabriele Marino,[2] Lee M. Butcher,[3] Gabriele Pollara,[1] Janos Kriston-Vizi,[4] Cristina Venturini,[1] Rachel Austin,[1] Karina Ferreira de Castro,[5] Robin Ketteler,[4] Benjamin Chain,[1] Richard A. Goldstein,[1] Robin A. Weiss,[1] Stephan Beck,[3] and Ariberto Fassati[1,6,*]

[1]Department of Infection, Division of Infection & Immunity, University College London (UCL), Cruciform Building, 90 Gower Street, London WC1E 6BT, UK
[2]Department of Veterinary Sciences, Polo Universitario dell'Annunziata, University of Messina, Messina 98168, Italy
[3]Department of Cancer Biology, Cancer Institute, UCL, 72 Huntley Street, London WC1E 6BT, UK
[4]MRC Laboratory for Molecular Cell Biology, UCL, Gower Street, London WC1E 6BT, UK
[5]Transmissible Cancer Group, Department of Veterinary Medicine, University of Cambridge, Madingley Road, Cambridge CB3 0ES, UK
[6]Lead Contact
*Correspondence: a.fassati@ucl.ac.uk
https://doi.org/10.1016/j.ccell.2018.03.003

SUMMARY

The canine transmissible venereal tumor (CTVT) is a clonally transmissible cancer that regresses spontaneously or after treatment with vincristine, but we know little about the regression mechanisms. We performed global transcriptional, methylation, and functional pathway analyses on serial biopsies of vincristine-treated CTVTs and found that regression occurs in sequential steps; activation of the innate immune system and host epithelial tissue remodeling followed by immune infiltration of the tumor, arrest in the cell cycle, and repair of tissue damage. We identified *CCL5* as a possible driver of CTVT regression. Changes in gene expression are associated with methylation changes at specific intragenic sites. Our results underscore the critical role of host innate immunity in triggering cancer regression.

INTRODUCTION

The canine transmissible venereal tumor (CTVT) is a contagious cancer allograft (Belov, 2012; Fassati and Mitchison, 2010; Murchison, 2008). Remarkably, CTVT is able to evade host immune-detection, allowing its worldwide spread in dogs (Belov, 2012; Murchison, 2008).

First described in the nineteenth century (Blaine, 1810; Novinski, 1876), the tumor grows mostly on male and female external genitalia and it is naturally transmitted between dogs by coitus, biting, or licking tumor-affected areas (Murchison, 2008). CTVT can be transplanted experimentally between dogs and even to other members of the *Canidae* family (Cohen, 1985). The similarity of the karyotype observed in CTVT samples from distant geographical areas suggested that this tumor originated from a common ancestor (Fujinaga et al., 1989; Idowu, 1977). A LINE element insertion into the MYC locus present in CTVTs but not in the host dogs supported this notion (Katzir et al., 1985). More recently, the clonal origin of CTVT was proven by analysis of microsatellite polymorphisms, mtDNA, and by dog leukocyte antigen (DLA) typing (Murgia et al., 2006; Rebbeck et al., 2009), and confirmed by genome-wide sequencing (Murchison et al., 2014). Together with the Tasmanian devil facial tumor disease (Belov, 2012) and the recently described leukemia-like cancer in soft-shell clams (Metzger et al., 2015, 2016), CTVT is a naturally occurring transmissible cancer of clonal origin.

CTVT was the first tumor to be experimentally transplanted before the era of inbred mice (Novinski, 1876). Experimentally transplanted CTVT is clinically characterized by a progressive (P), a stationary (S), and a regressive (R) phase (Epstein and Bennett, 1974). In the P phase, there is rapid growth of the tumor to become a pedunculated, cauliflower-like exudative mass.

Significance

There are three known clonally transmissible cancers in nature: CTVT, the Tasmanian devil facial disease, and leukemias of clams. CTVT is the only one that regresses, spontaneously or after vincristine administration, providing a unique model for cancer regression in general. By performing systematic genome-wide analysis of CTVT regression, we found that treatment with vincristine induces host innate immune genes and epithelial differentiation, triggering immune-rejection of the tumor. Gene expression changes correlate with demethylation of specific intragenic regions. Our results provide a unique insight into the molecular and immunological mechanisms driving cancer regression and support recently proposed models whereby innate immunity plays a critical role in triggering cancer rejection.

 © 2018 The Author(s). Published by Elsevier Inc.
This is an open access article under the CC BY license (http://creativecommons.org/licenses/by/4.0/).

Table 1. Histopathology on Tissue Sections of CTVT-5 and CTVT-6

CTVT Sample, Day (Biopsy Number)	Sex of Dog	CTVT Phase	Mitosis/1.000 Cells	Lymphocytes (%)	Granulocytes (%)	Apoptotic/Necrotic Cells (%)
5, day 0 (biopsy 1)	male	P	2.4	3.40	2.70	0
5, day 6 (biopsy 2)		S/R	0	15.60	NA	16.30
5, day 14 (biopsy 3)		R	0	NA	NA	80
6, day 0 (biopsy 1)	female	P	4.7	0	8.86	0
6, day 6 (biopsy 2)		P/S	0	1	0	20
6, day 14 (biopsy 3)		S	0	1	0	20
17[a] day 0 (biopsy 1)	male	P	NA[b]	NA	NA	NA
17[a] day 22 (biopsy 2)		S	NA	NA	NA	NA
17[a] day 48 (biopsy 3)		S/R	NA	NA	NA	NA

[a]CTVT-17 could not be analyzed due to poor tissue preservation.
[b]NA, not available.
See also Figure S1.

Microscopically there is abundance of mitotic cancer cells and few infiltrating lymphocytes. In the S phase, growth slows considerably; there are fewer cancer cells in mitosis, and more apoptotic cells and infiltrating lymphocytes. In the R phase, there are abundant infiltrating lymphocytes, cancer cells disappear, the tumor stroma collapses, and there is collagen deposition (Chu et al., 2001; Mukaratirwa et al., 2004). Recovered dogs are immune to re-inoculation (Cohen, 1985). In naturally occurring CTVT, spontaneous regression is also observed, albeit less frequently than in transplanted CTVT (Perez et al., 1998). However, natural CTVT is often sensitive to radiotherapy and chemotherapy and even a single treatment may induce regression (Gonzalez et al., 2000; Thrall, 1982).

Histology of tumors in the P and R phases indicated that regression is characterized by apoptosis of cancer cells and the presence of tumor-infiltrating lymphocytes (TILs) (Gonzalez et al., 2000; Perez et al., 1998) with production of anti-tumor immunoglobulin Gs (Epstein and Bennett, 1974). Thus complete regression seems to depend on an appropriate immune response, a notion supported by the fact that immunosuppressed dogs and puppies develop more aggressive CTVT that lacks TILs and is rarely eliminated (Yang and Jones, 1973).

It is not clear how CTVT evades immune-detection during transmission and growth but triggers rejection in the R phase. In transplanted CTVT, a model has been proposed whereby secretion of transforming growth factor β (TGF-β) by cancer cells suppresses class I and II DLA expression and NK (natural killer) cell activity. TGF-β is counteracted by interleukin-6 (IL-6), produced by TILs, hence, when a critical threshold is reached, TGF-β is overcome by IL-6, resulting in re-expression of DLAs on CTVT cells and their rapid elimination (Chiang et al., 2013; Hsiao et al., 2004). There is little evidence, however, that this mechanism operates in natural CTVT.

Although the natural spread of CTVT is confined to dogs, humans and dogs share many forms of cancer with similar clinical presentation, pathology, and genetic mutations, including osteosarcoma, soft tissue sarcoma, non-Hodgkin lymphomas, and melanoma (Schiffman and Breen, 2015). This suggests that understanding regression of CTVT could be important in identifying potential mechanisms of regression in human cancers. To understand the process leading to CTVT regression, we analyzed biopsies from natural CTVTs collected before and after treatment with vincristine and contrasted cases that fully regressed to cases that did not regress.

RESULTS

We collected three serial biopsies from two naturally occurring tumors, one in a male (CTVT-5) and one in a female (CTVT-6) mixed breed dog (Table 1). The dogs were treated with a single intravenous administration of 0.025 mg/kg vincristine. The first biopsy was collected pre-therapy; the second and third biopsies were collected 6 and 14 days post-therapy, respectively. Macroscopically, both pre-therapy CTVTs were large (>3 cm diameter), cauliflower-like, ulcerated, and bleeding (Figure S1). Both CTVTs responded to vincristine, becoming smaller with reduced bleeding. However, at the time of the third biopsy, CTVT-5 had almost completely regressed, whereas CTVT-6 still presented a significant tumor mass (Figure S1). Biopsies were examined microscopically to count mitotic figures, TILs, granulocytes, and apoptotic cells (Table 1). The most striking difference between the two samples was the lack of TILs in CTVT-6, whereas in CTVT-5 TILs represented 3.4% of total cell count in the first biopsy and 15.6% in the second biopsy. Both samples contained 15%–20% of apoptotic cells in the second biopsy, increasing to >80% in CTVT-5 but remaining stable in CTVT-6. Based on the macroscopic appearance and the histology, we classified the CTVT-5 serial biopsies as P, S, and R phases and the CTVT-6 biopsies as P, P/S, and S, respectively. A third case in a male mixed breed dog (CTVT-17) (Table 1; Figure S1) presented at a different clinic after the first two cases and a biopsy was collected at day 0, and days 22 and 48 post-vincristine. Clinical examination indicated that the biopsy taken at day 22 was S phase and biopsy taken at day 48 was R phase. Poorer tissue preservation prevented pathological analysis of CTVT-17.

RNA-Seq Identifies Genes Differentially Regulated in S and R Phases

To gain a deeper understanding of the mechanisms of CTVT regression, we sought to identify specific genes and pathways that are differentially expressed in the serial biopsies. We reasoned that genes driving regression would be differentially

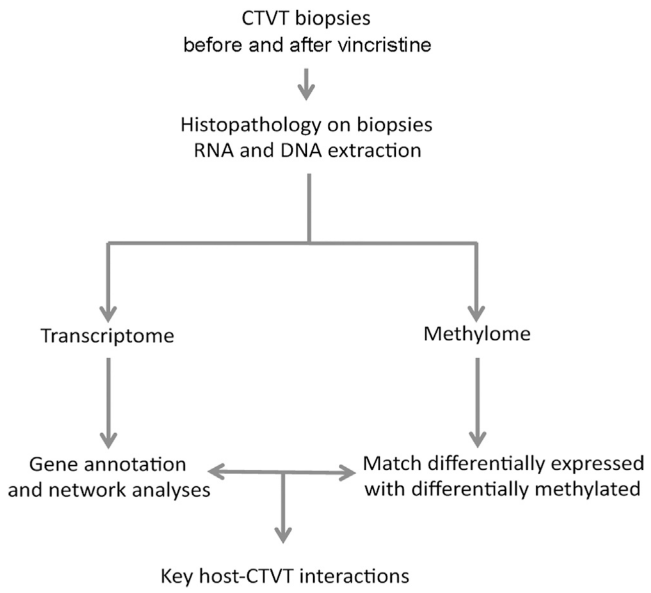

Figure 1. Diagram of the Experimental Pipeline Used to Study CTVT Regression

regulated in P versus S or R phase. We also sought to determine if epigenetic regulation of gene expression might be important. Therefore, we performed transcriptome and methylome analyses in parallel to match differentially regulated and differentially methylated genes and conducted gene pathway analysis to identify key host-CTVT interactions. CTVT-6 provided an important control because it did not regress. Our approach is summarized in Figure 1.

Nucleic acids were extracted from biopsies and total RNA was prepared for sequencing following the Illumina TruSeq mRNA protocol and sequenced on an Illumina NextSeq to yield an average of 15 million reads per sample. RNA extracted from CTVT-17 did not pass quality control and could not be sequenced. Following alignment and removal of PCR duplicates, we applied rigorous generalized linear models for all of the differential gene expression analyses, modeling count data using the negative binomial distribution via the BioConductor library DESeq and using a matrix (regression status, CTVT, time point before and after treatment), which allows to incorporate paired data into multifactorial analysis (Anders and Huber, 2010; Gentleman et al., 2004) (Table S1). We compared changes in gene expression occurring in each sequential biopsy; a total of 6,756 genes were found differentially regulated (2-fold or more). To better capture changes likely to be of biological significance, we applied two stringent cutoffs, requiring a fold-change (FC) > 10 and an adjusted p value (adj-p) < 0.01 (Benjamini-Hochberg test). Using this filter, we detected 1,016 differentially expressed genes, of which 704 were upregulated and 312 downregulated (Table S1).

Expression analysis (Figure 2A; Table S2) revealed that some genes were already upregulated in the second biopsy, while others were upregulated in the third biopsy. For CTVT-6, both the first and second biopsies were more similar to the P phase of CTVT-5 than to the S phase, whereas the third biopsy was more similar to the S phase of CTVT-5 (Figure 2A). Downregulated genes progressively faded in CTVT-5 but not in CTVT-6 (Figure 2A). These findings agreed with the lack of clinical regression and the pathology of CTVT-6 (Table 1; Figure S1).

To confirm and extend the RNA sequencing (RNA-seq) results, we investigated six additional CTVT cases (CTVT-7xx) acquired by natural transmission, three of which regressed (CTVT-765, -766, and -772) and three of which did not (CTVT-761, -774, and -775) following treatment with vincristine. Due to ethical and practical constraints, biopsies from these CTVTs were collected before treatment (biopsy 1 or B1) and at day 28 after vincristine (biopsy 2 or B2); hence they did not have the same temporal resolution of CTVT-5 and CTVT-6. Nonetheless, these samples were collected years apart from CTVT-5 and CTVT-6, on a different continent and were sequenced in a different laboratory, and thus can be considered as a completely independent experiment to validate key results.

To confirm the clinical observations, DNA from these 7xx CTVTs was analyzed by qPCR to measure the LINE-Myc insertion, a hallmark of this tumor (Katzir et al., 1985), as well as a region of MHC class II *DLA DQA-1* shared between CTVT and host and a region of *DLA DQA-1* specific for CTVT (Murgia et al., 2006). CTVT-765, -766, and -772 regressed clinically and showed loss of LINE-Myc DNA and tumor-specific *DLA DQA-1*. In contrast, CTVT-761 and -774 did not regress clinically and maintained LINE-Myc and tumor-specific *DLA DQA-1* (Figure 2B). CTVT-775 also did not regress clinically but showed some loss of LINE-Myc and tumor-specific *DLA DQA-1* (Figure 2B).

RNA from these samples was sequenced to yield between 18 and 31.5 million reads per sample, except B 1 of CTVT-774, which did not pass quality controls and was not included in the subsequent analysis. Principal-component analysis revealed a clear separation between B1 and B2 of the 7xx regressing tumors but not for the biopsies of non-regressing CTVTs (Figures S2A and S2B). To examine the overall reproducibility of the experiments, we compared $\log_2$FC in gene expression in B3/B1 of CTVT-5 with the mean $\log_2$FC in B2/B1 of the regressive or non-regressive 7xx CTVTs. Considering all expressed genes, correlation (Pearson's coefficient) was 0.80 for the regressive CTVTs (Figures S2C and S2D), rising to 0.94 when considering only those genes that passed the FC > 10 adj-p < 0.01 cutoff, indicating high reproducibility.

In the regressive 7xx CTVTs, 1,350 genes were differentially regulated in B2 relative to B1 (FC > 10 and adj-p < 0.01), but only 59 genes met these cutoffs in the non-regressive 7xx CTVTs (Table S1). Expression analysis of these 1,350 genes revealed a pattern of expression changes similar to CTVT-5 and CTVT-6, which discriminated between regressing and non-regressing tumors (Figure 2C). The overlap between significantly changed genes in CTVT-5 (B3/B1) and in the regressive 7xx CTVTs (B2/B1) was 63.7% (648/1,016 genes) (Figure 2D). Further analysis of the 1,350 genes significantly changed in the regressive 7xx CTVTs identified 189 genes that were also statistically significantly different ($p < 0.05$) when comparing changes in their expression levels (B2/B1) in regressive versus non-regressive 7xx CTVTs (regressive [R]-B2/R-B1 versus non-regressive [NR]-B2/NR-B1) (Figure 2C, red lines on the right; Table S2). Of these 189 genes, 127 genes were also differentially expressed

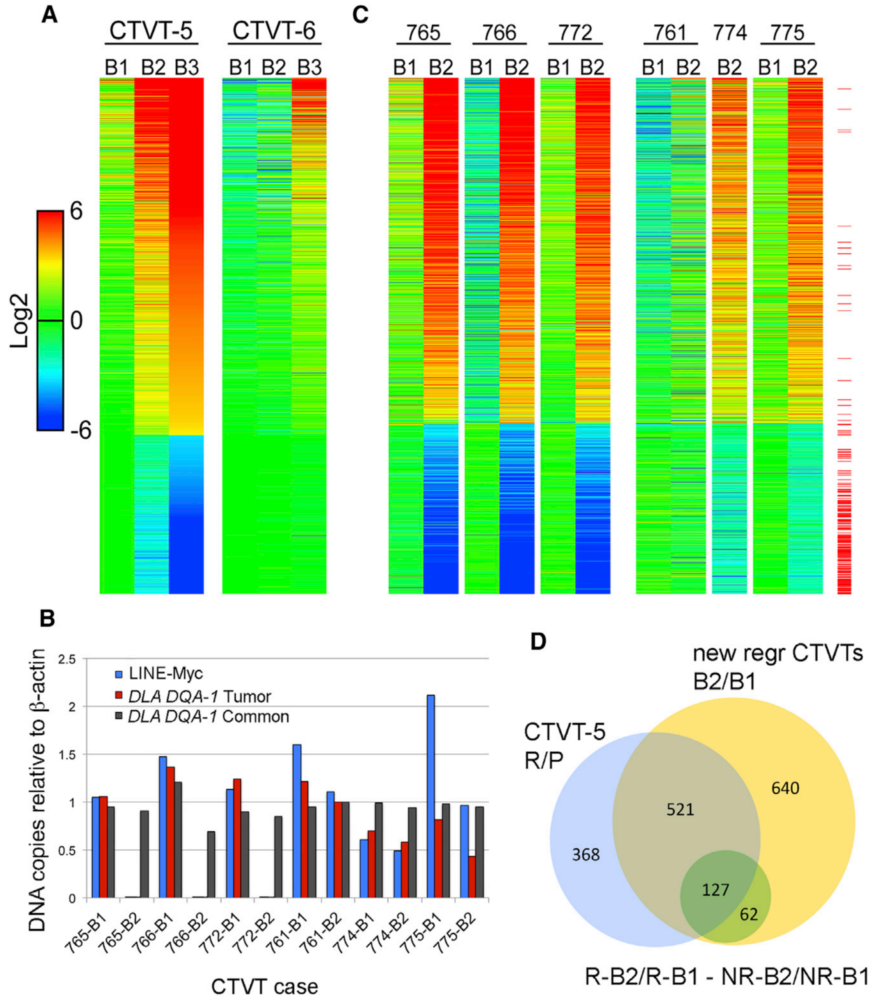

Figure 2. Transcriptional Profiling Detects a Signature of CTVT Regression

(A) Heatmap showing differential expression of the 1,016 significant genes (FC > 10 and adj-p < 0.01) relative to the geometric mean of CTVT-5 B1 and CTVT-6 B1 (mean of the logs). Genes are sorted by expression of B3/geometric mean of (CTVT-5 B1, CTVT-6 B1). B1, first biopsy; B2, second biopsy; B3, third biopsy.

(B) qPCR on DNA extracted from biopsies obtained before (B1, day 0) and after (B2, day 28) vincristine administration of the 7xx CTVTs. Specific primers were used to amplify LINE-MYC DNA (tumor specific), *DLA DQA-1* (tumor specific), and *DLA DQA-1* (common to both host and tumor). All quantifications are relative to β-actin.

(C) Heatmap showing differential expression of the 1,350 significant genes in the 7xx CTVTs (FC > 10 and adj-p < 0.01) relative to the geometric mean of the five B1 samples. Genes are sorted by expression of B2/geometric mean of 7xx B1s. Each row represents an individual gene. RNA-seq data were not available for CTVT-774 B1. The red lines next to each row indicate genes that reached statistical significance in the R-B2/R-B1 versus NR-B2/NR-B1 comparison.

(D) Venn diagram showing the degree of overlap between CTVT-5 R versus P genes (1,016 genes), the 7xx CTVTs B2 versus B1 genes (1,350 genes) and genes that reached statistical significance in the R-B2/R-B1 versus NR-B2/NR-B1 comparison (189 genes).

See also Figure S2 and Tables S1 and S2.

(FC > 10 adj-p < 0.01) in CTVT-5 but not in CTVT-6, thus forming a potential "core" signature of regression: they reproduce across experiments and are statistically significantly different between regressing and non-regressing tumors within the second experiment (Figure 2D; Table S2). Overall, these data indicated the existence of a signature of regression, which we sought to explore in greater depth.

Inflammation and Epithelial Cell Proliferation Characterize the Early Response to Vincristine

The CTVT-5 expression analysis revealed dynamic changes in gene expression and we hypothesized that this may reflect sequential steps ultimately leading to CTVT regression. We therefore examined the temporal nature of the changes in gene expression (Figure 3A). We classified genes into several groups based on when they reached an FC > 10 (at the adj-p < 0.01 cutoff): we found 150 "early up" genes (upregulated [>10-fold] in S phase relative to P phase), 203 "late up" or "late down" genes (up- or downregulated [>10-fold] in R relative to S, but not in S relative to P), and 663 "progressive" genes (progressively up- or downregulated [>10-fold] in R relative to P, but not in R relative to S, or S relative to P) (Figures 3A and 3B).

Differentially expressed genes corresponding to these three different temporal profiles were studied in each CTVT for pathway enrichment using Ingenuity Pathway Analysis (IPA) (www.qiagen.com/ingenuity) (Figures 3C–3E). Individual IPA profiles were compared based on statistical significance (Fisher's exact test) and similarity (Figure 3C; Table S3). In CTVT-5, the top early networks were related to recruitment of, and invasion by, granulocytes, agranulocytes, and leukocytes, formation and inflammation of skin, as well as a broader "cancer" signature. This was followed by proliferation and activation of lymphocytes, NK cells, and B cells. The late stage was characterized by upregulation of networks related to migration and invasion of cells in general, proliferation of connective tissue, and migration of vascular endothelial cells suggesting formation of new tissue (Figures 3C–3E; Table S3). These pathways were shared between CTVT-5 and the regressive 7xx CTVTs (Figures 3C–3E; Table S3).

IPA showed an enrichment within the early up gene group for genes involved in skin inflammation, epithelial cell differentiation and development, and cell migration (p < 0.01, Fisher's exact test) (Table S3). Key genes in these early networks that were also differentially expressed in the regressive 7xx CTVTs (FC > 10 and adj-p < 0.01; Tables S1 and S2) included *TP63*, which is required for epithelial and keratinocyte differentiation (Crum and McKeon, 2010; Stramer and Martin, 2005), as well as keratins such as *KRT4* (FC = 92) and *KRT15* (FC = 104), which

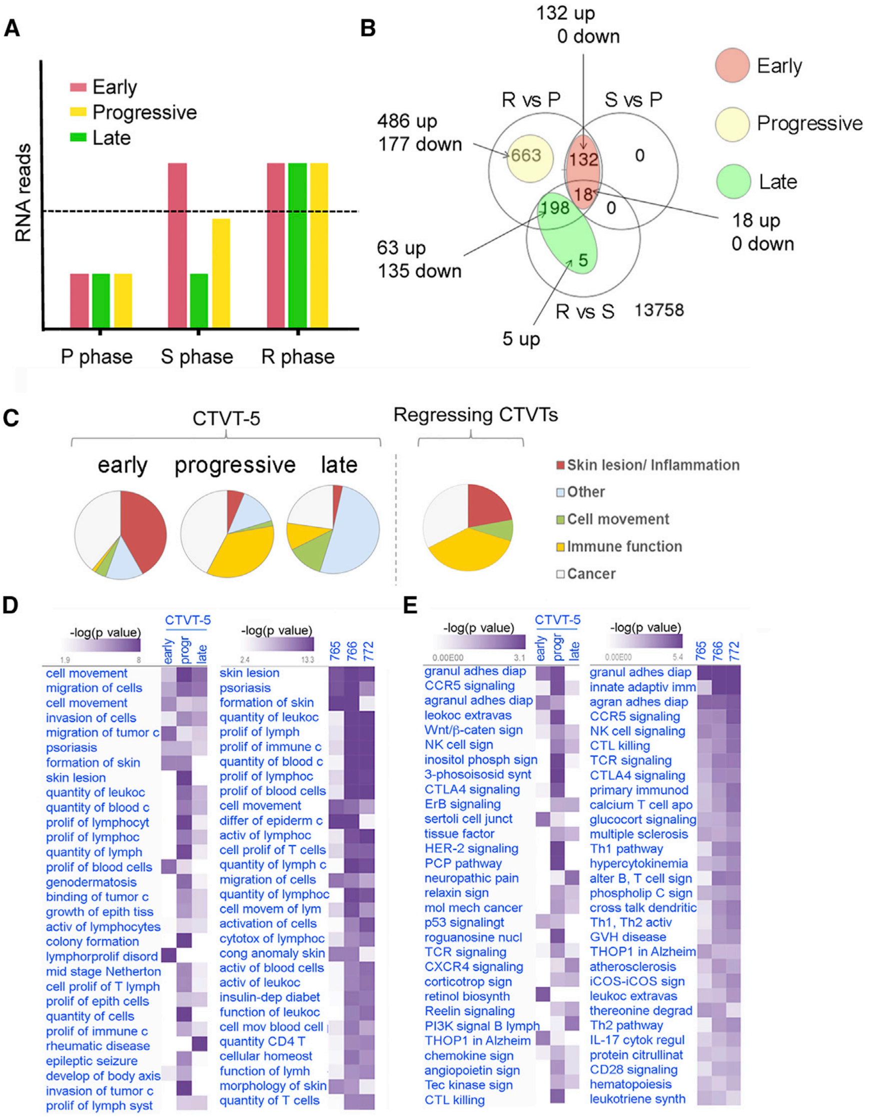

Figure 3. Dynamic Changes in Gene Expression across Sequential Biopsies Identify Pathological Processes during CTVT Regression

(A) Schematic depiction of the changes in expression for the early, progressive, and late genes in the different CTVT phases. The dotted line indicates an expression FC > 10 relative to baseline values in the P phase.

(B) Venn diagram illustrating the overlap between differentially expressed genes across all three comparisons. The diagram explains how differentially expressed genes were classified into three categories: early, late, and progressive.

(C) Pie charts indicating the relative proportion of IPA gene networks within the early, progressive, and late upregulated gene groups of CTVT-5 and their relative proportion within the regressing 7xx CTVTs.

(D and E) Heatmaps of comparative IPA analyses showing the top 30 diseases and biofunction pathways (ex cancer) (D) or canonical pathways (E) for B1, B2, and B3 of CTVT-5 and each individual regressive 7xx CTVTs. Pathways are clustered based on significance (−log10 p value, Fisher's exact test) and similarity.

See also Table S3.

are expressed in the basal layer of stratified epithelia (Moll et al., 2008) (Figure 4A). Overall, within this group, the overlap between CTVT-5 and the regressing 7xx CTVTs was 78% (42/54 genes) (Table S4). There was a prominent skin inflammatory component in the network, classified by IPA as psoriasis and Netherton syndrome, a disease characterized by chronic inflammation and dermal infiltrate of eosinophils and neutrophils (Leclerc-Mercier et al., 2016) (Figure 4A). Key early up genes involved in inflammation included *S100A8* (FC = 18) and *S100A9* (FC = 14), which are endogenous TLR4 ligands (Vogl et al., 2007), *PIGR* (FC = 560), which mediates transport of IgA across mucosal epithelial cells (Kaetzel, 2005), as well as the chemotactic cytokine *CCL5* (FC = 11) (Figure 4A; Table S1).

To identify possible early drivers of regression, we plotted normalized RNA reads of early up genes of CTVT-5, which regressed, versus CTVT-6, which did not regress. *CCL5* was among the most prominent outliers (Figure 4B). CCL5/RANTES is a key chemokine, which, by binding to its receptor CCR5, promotes chemotaxis of monocytes, T lymphocytes, NK cells, eosinophils, and basophils, and can be produced by epithelial cells and keratinocytes (Li et al., 1996; Proost et al., 1996). *CCL28* (FC = 30 in CTVT-5 and 3 in CTVT-6) was another prominent outlier (Figure 4C; Table S1). These chemokine genes also reached higher expression levels in the regressive relative to the non-regressive 7xx CTVTs and high absolute levels (Figure 4D). The relative change in *CCL5* expression was significantly different between regressive and non-regressive CTVTs (R-B2/R-B1 versus NR-B2/NR-B1, $p < 0.01$), whereas *CCL28* reached statistical significance in the regressive CTVTs only (Figure 4D). CCR5 signaling was among the most significant canonical pathways identified by IPA in the regressive 7xx CTVTs (Figure 3E; Table S3). Furthermore, *CCL5* was one of four early up genes in the core signature of regression, the others being *CD8*, *MFAP4*, which also promotes monocytes chemotaxis (Schlosser et al., 2016), and *UBASH3A*, a member of the T cell ubiquitin ligand family (Table S2). Thus *CCL5* might be important to trigger rejection.

Immune Cell Infiltration, Cell-Cycle Arrest, and Tissue Remodeling Characterize the Secondary Response to Vincristine

Next we focused on the prominent immunological network detected by IPA in all the regressive CTVTs. A group of 88 immunological genes was significantly upregulated in CTVT-5, but not in CTVT-6, with a good overlap of individual genes between CTVT-5 and the regressive 7xx CTVTs (≈60% overlap at

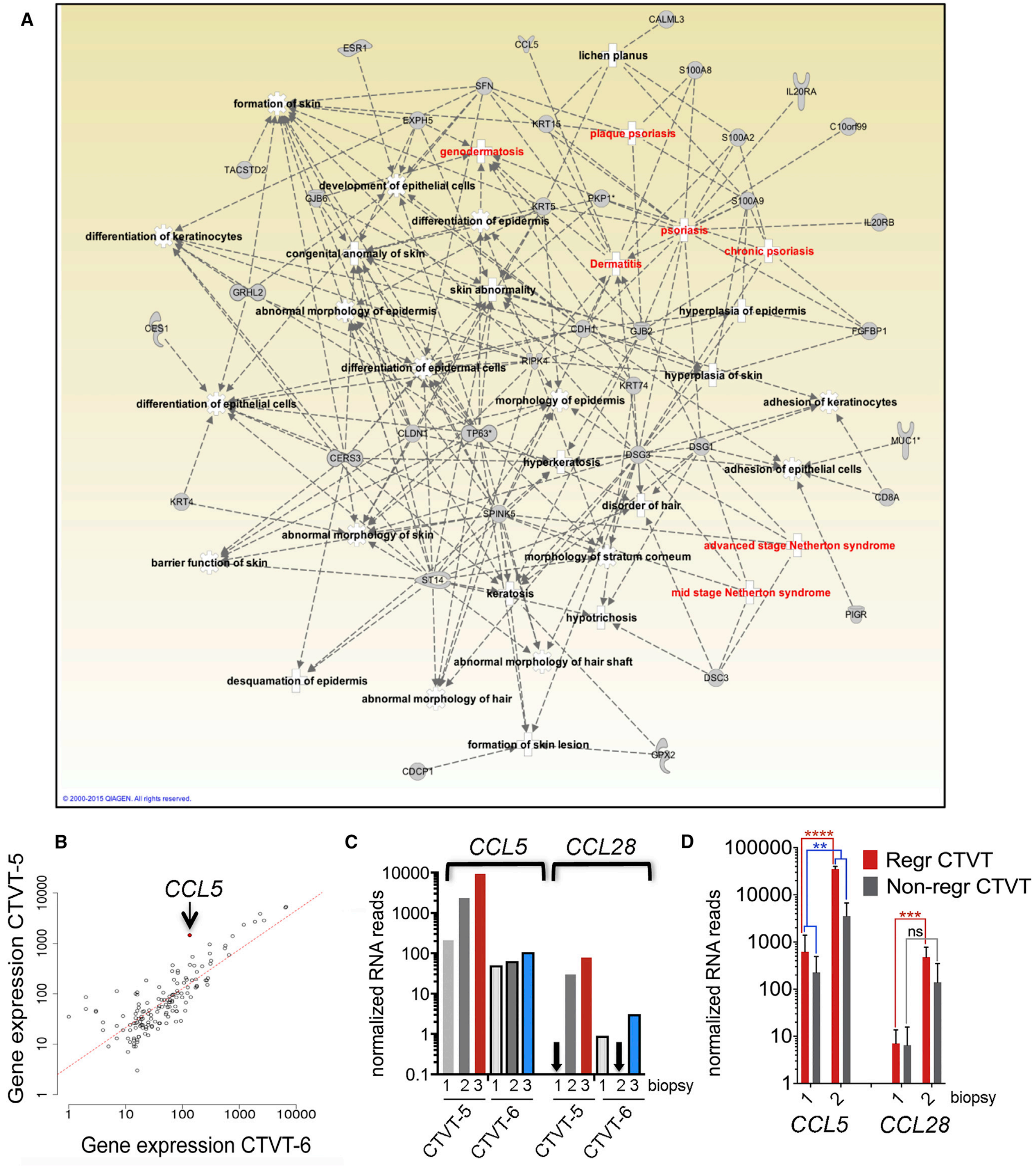

Figure 4. Early Upregulation of Epithelial and Inflammation-Related Genes Characterizes CTVT Regression

(A) IPA diagram showing gene networks of early upregulated genes in CTVT-5. Inflammation-related nodes are highlighted in red. (B) Scatterplot illustrating correlation between normalized RNA-seq counts for early upregulated genes in CTVT-5 (B2) and CTVT-6 (B3). Best-fit line is shown in red.

(C and D) Gene expression levels of *CCL5* and *CCL28* in CTVT-5 and CTVT-6 (C), and in the additional 7xx CTVT samples (D) (mean ± SEM, n = 3). Significant adj-p values are shown (****$p < 0.0001$, ***$p < 0.001$, **$p < 0.01$): all have FC > 10.

See also Table S4.

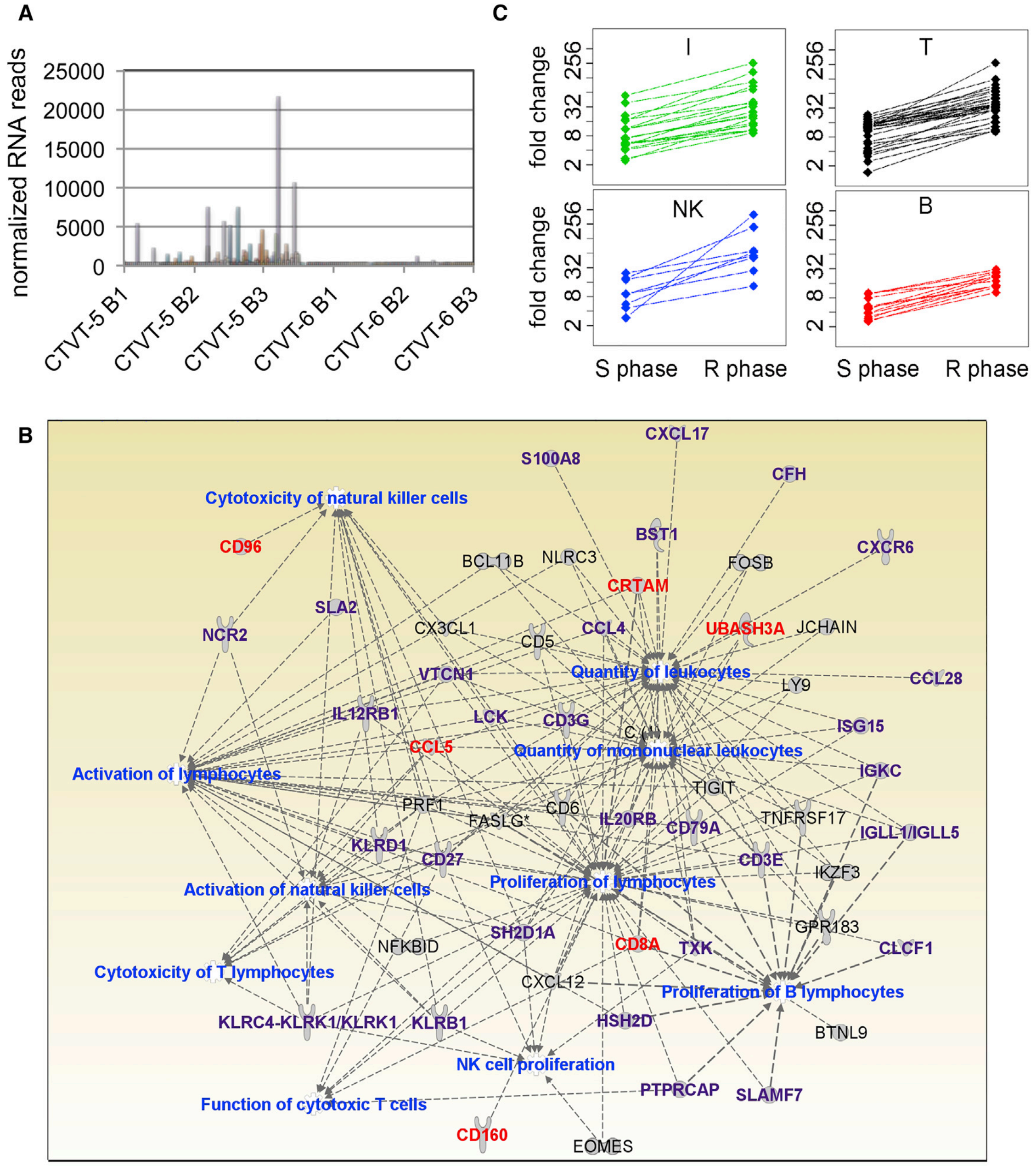

Figure 5. Upregulation of T, NK, and B Cell-Related Genes Characterizes the Secondary Response to Vincristine

(A) Expression changes in B1, B2, or B3 of 88 progressive immunological genes in CTVT-5 and CTVT-6.

(B) Diagram showing 10 representative nodes obtained by IPA using the 88 progressive immunological genes upregulated in CTVT-5. Key nodes are shown in blue; key genes belonging to the core signature of regression are shown in red; progressive up genes in common between CTVT-5 and the regressive 7xx CTVTs are shown in purple.

(C) The 88 immunological genes were annotated manually using Genecards and the available literature into four groups: I, inflammation/innate immunity; T, T cells; NK, natural killer cells; and B, B cells. Fold upregulation of genes in the S and R phase is relative to the P phase and is based on the normalized RNA-seq reads.

See also Tables S4, S5, and S6.

FC $\geq$ 10 and adj-p $<$ 0.01) but a 0% overlap with the non-regressive CTVTs at the same cutoff (Figure 5A; Table S4). These genes formed networks, which included CCR5 signaling, leukocyte extravasation, and T, NK, and B cell function (Figure 5B; Table S3).

We classified these immunological genes into four groups based on the existing literature and available experimental evidence: genes involved in inflammation, T cell function, NK cell function, and B cell quantity or function (Figure 5C; Table S4). Inflammation-related genes were the most upregulated in the

S phase and were further upregulated in the R phase. T cell- and NK cell-related genes were progressively upregulated. B cell genes, albeit less prominent in quantity and expression levels, were also progressively upregulated (Figure 5C), in agreement with pathological studies reporting infiltration of CTVT by B cells (Perez et al., 1998) and the presence of a B cell signature in acute allograft rejection (Sarwal et al., 2003).

To further validate key pathways, we performed IPA using the 189 genes that reached statistical significance in the R-B2/R-B1 versus NR-B2/NR/B1 comparison for the 7xx CTVTs or the 127 genes forming the core regressive signature (Figure 2D; Table S2). We compared these IPAs with those obtained with each regressive CTVT. This analysis confirmed that granulocyte/agranulocyte adhesion and diapedesis, communication between innate and adaptive immunity, T cell signaling, and CCR5 signaling were the most significant upregulated pathways across all datasets (Table S5).

To better understand the contribution of the host and the tumor to the changes in gene expression, we took advantage of several sources of information regarding tissue origin. Firstly, deep sequencing of the CTVT genome revealed that 657 genes were deleted or had a premature stop codon in CTVT (Murchison et al., 2014) (Table S1), hence they could be used as markers of host tissue. Secondly, the CTVT genome has accumulated over two million mutations (Murchison et al., 2014), which we could use to map a particular gene to host or CTVT, provided that the sequencing depth was sufficient and that at least two mutations were present within a particular transcript. Using this filter, we sampled 63 upregulated genes that passed the FC > 10 adj-$p < 0.01$ cutoff in CTVT-5 and were also involved in key IPA networks, including skin inflammation. The analysis showed that almost all of them were of host origin (Table S6), indicating that the host stroma contributes to CTVT regression.

We found no early downregulated genes and instead found 177 progressive genes downregulated in CTVT-5 relative to CTVT-6. The main functional networks affected were cell cycle, DNA replication and recombination, and organ development (Figure 6A; Table S3). The large cell-cycle network included many genes involved in the formation of the mitotic spindle, condensation, and segregation of chromosomes (Figures S3A and S3B; Table S7). This is consistent with the inhibitory effect of vincristine on microtubule dynamics, leading to perturbation of the mitotic spindle and cell-cycle arrest (Ngan et al., 2001). These genes remained unchanged in CTVT-6, confirming the specificity of the effect (Figure S3A; Table S7). Notably, the cell-cycle gene network was not detected in the regressive 7xx CTVTs (Figure 6A; Table S3), suggesting that the dramatic changes in cell cycle occurred early and were missed in the second biopsy of the regressive 7xx CTVTs, which was collected 28 days after vincristine treatment.

Pathway Analysis on Genes that Cease to Be Expressed during Regression Suggests that CTVT May Be Similar to Melanoma

The late downregulated genes were only detected in CTVT-5 and not in CTVT-6, suggesting that they were closely linked to advanced tumor regression (Table S1). The third biopsy of CTVT-5, collected at 14 days after vincristine administration, contained 80% necrotic or apoptotic cells (Table 1). We took advantage of this fact to investigate the possible cell or tissue of origin of CTVT. We reasoned that genes that disappeared in the last biopsy were more likely to belong to CTVT (except for immunological genes), and hence could be used to obtain a transcriptional profile of the tumor itself. There were 135 genes downregulated in the late stage. The IPA functional networks with the higher confidence were related to solid cancers (Figure 6B), and within these groups melanoma had the highest confidence and a large number of genes (80 genes, $p = 1.22 \times 10^{-8}$) (Figures 6B and S4; Table S3). Remarkably, IPA of downregulated genes in the regressive 7xx CTVTs also showed that the functional networks with the highest confidence and greatest number of genes were skin cancer and cutaneous melanoma (>180 genes, $p < 1.52^{-17}$) (Figure 6B; Table S3). The top three IPA disease pathways identified across all CTVTs were skin cancer, cutaneous melanoma, and melanoma (Figure 6C). Furthermore, skin cancer and cutaneous melanoma emerged as the most significant downregulated disease and biofunctions pathways within the core signature of 127 genes and across all datasets (Table S8).

Although the highly significant cancer signature was expected, the melanoma signature was not, given that CTVT has been proposed have a histiocytic origin (Cohen, 1985; Mozos et al., 1996). The histological classification of CTVT was mainly based on positive immunostaining for lysozyme (encoded by *LYZ*), α1-antitrypsin (encoded by *SERPINA1*), and vimentin (encoded by *VIM*) in about 30%–50% of the tumor cells (Mozos et al., 1996). However, our RNA-seq analysis (Table S1) showed no significant change in the expression levels of *LYZ* (Ensembl ID: ENSCAFG00000000426), *SERPINA1* (Ensembl ID: ENSCAFG00000017646), or *VIM* (Ensembl ID: ENSCAFG00000004529) in any regressing CTVTs, hence it is unlikely that such markers are specific for the tumor, although we note that histamine biogenesis was a prominent canonical pathway (Figure 6D).

To explore this issue further, we assessed the relative enrichment of our late downregulated gene signature in the human NCI-60 cancer cell line panel originating from other solid tumors (Pfister et al., 2009). We found that melanoma was among the most enriched tumor cell types, and that this showed the most significant difference when compared with all other solid tumors in the panel (Figures S4B and S4C). Thus we concluded that CTVT has similarities to melanoma at the transcriptional level.

DNA Methylation Changes during CTVT Regression

Next we investigated whether changes in gene expression were associated with epigenetic modifications. DNA methylation at CpG islands in promoters often causes silencing of genes and, conversely, their demethylation stimulates gene expression (Bird, 1986; Esteller, 2005). However, it was also shown that methylation occurs in other DNA regions, such as CpG island shores within 2 kb of transcriptional start site and even within exons near the 3′ end of genes (Hovestadt et al., 2014; Li et al., 2010). Methylation of such regions appears to impact on gene expression more strongly than CpG island methylation (Hovestadt et al., 2014). Therefore, we conducted a global methylation analysis using MeDIP-seq (Taiwo et al., 2012) on the sequential biopsies of CTVT-5, CTVT-6, and CTVT-17, and mapped methylation levels within the promoter, the first exon,

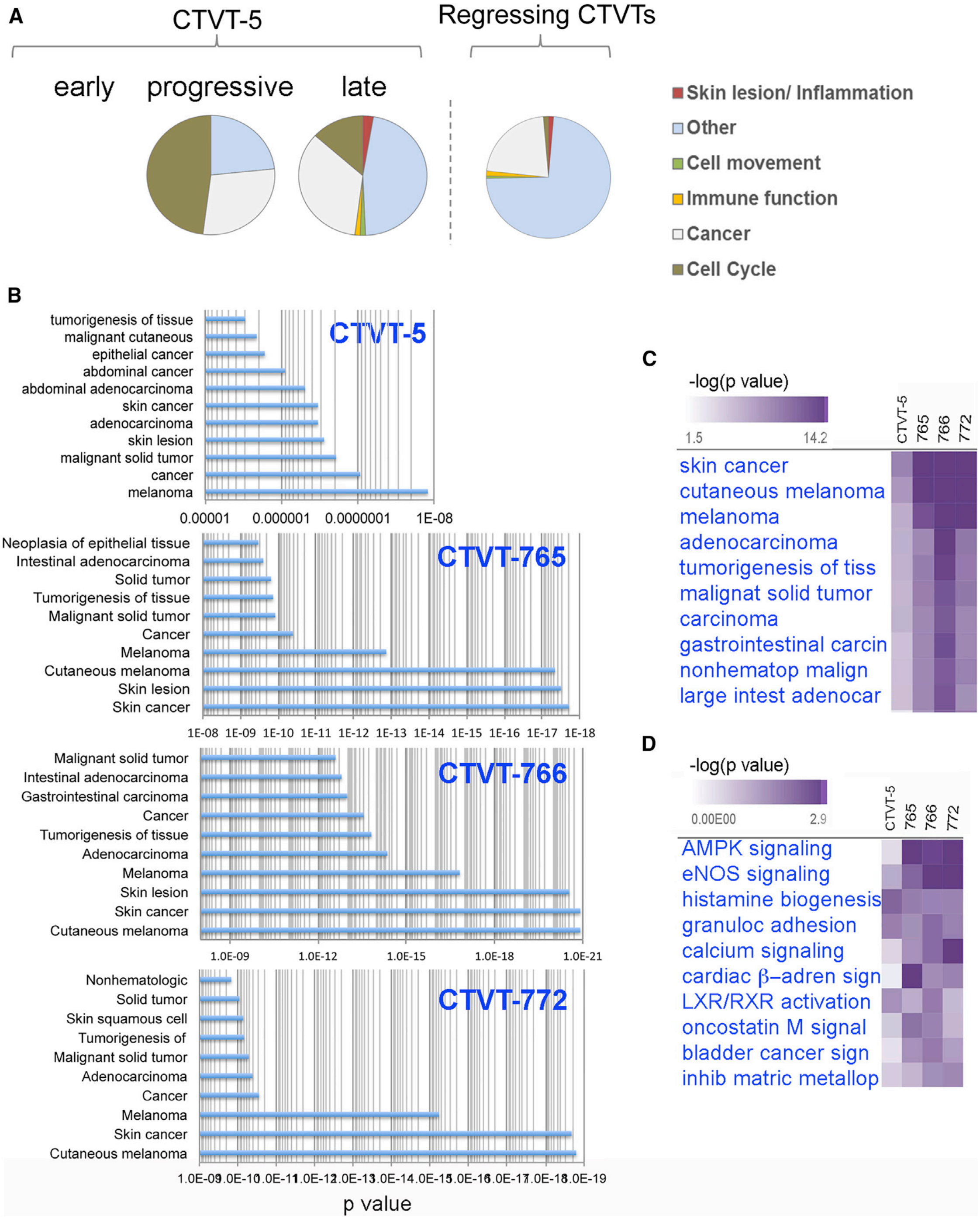

Figure 6. Downregulated Genes Show a Melanoma-like Signature

(A) Pie charts indicating the relative proportion of each functional IPA network within the progressive and late downregulated gene groups of CTVT-5 and within the downregulated gene groups of the regressing 7xx CTVTs. Note that no early downregulated gene was detected in CTVT-5.

(B) Plots showing the confidence value of the main functionally annotated pathways identified by IPA for the late downregulated gene group in each regressing CTVT. The p value (Fisher's exact test) for each pathway is shown on the x axis.

(C and D) Heatmaps of comparative IPA analyses showing the top 10 diseases and biofunction pathways (C) or canonical pathways (D) for each individual regressive CTVTs. Pathways are clustered based on significance (Fisher's exact test) and similarity.

See also Tables S7 and S8; Figures S3 and S4.

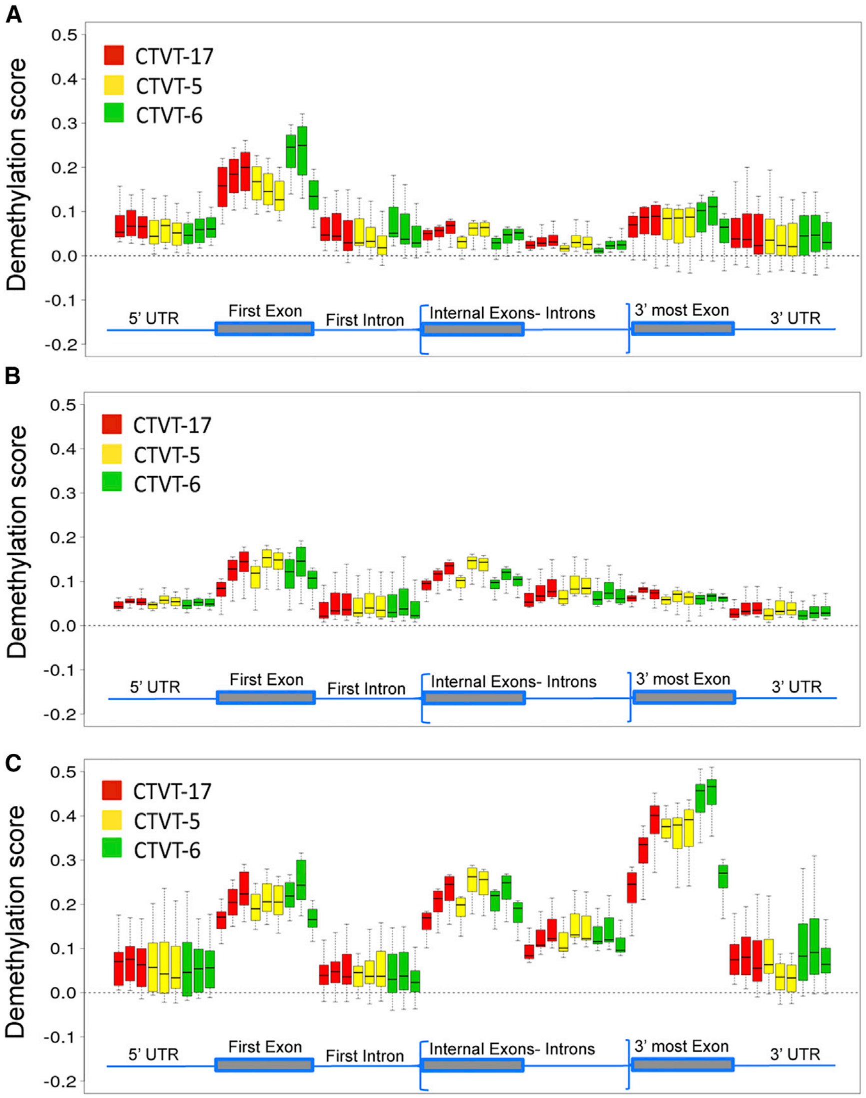

Figure 7. Changes in Gene Expression Correlate with Specific Changes in DNA Methylation

Demethylation profiles of early (A), progressive (B), and late (C) upregulated genes across serial biopsies of CTVT-17 (red), CTVT-5 (yellow), and CTVT-6 (green). Demethylation scores were obtained for individual genes by quantifying demethylation levels within specific regions of genes (≤2 kb upstream of first exon; first exon; first intron; internal exons; internal introns; last exon; ≤2 kb downstream of last exon) and normalized by subtracting the corresponding demethylation values observed for non-expressed genes. Boxplots illustrate the variation within these values across each gene-list (boxes extend to the first and third quartile, whiskers extend to 1.5× interquartile range, and the line represents median values). For each CTVT sample, boxplots are in order (from left to right): first, second and third biopsy.

See also Figure S5.

the first intron, internal exons, internal introns, the last exon, and the 3′ end of genes. We then matched the methylation pattern for each gene with our transcriptional data (RNA-seq) to correlate methylation levels and location to gene expression. We maintained the same overall classification based on when genes were up- or downregulated (early, progressive, and late). To detect specific changes, we normalized the signal by subtracting the methylation values of genes that were not expressed in any of the biopsies. Although we were unable to perform transcriptional analysis on CTVT-17, we observed consistent methylation profiles across all biopsies for each gene list obtained from RNA-seq of CTVT-5 and CTVT-6 (Figures 7, S5A, and S5B).

In the early upregulated genes we observed demethylation at the first exon across all CTVT samples. This demethylation pattern was sustained in CTVT-5 and CTVT-17, but was reduced in the third biopsy of CTVT-6 (Figure 7A). A more complex demethylation pattern was observed in the late upregulated genes. Here internal exons and the last exon showed either progressive or sustained demethylation, from the first to the last biopsy. The exception was CTVT-6, whose demethylation levels dropped in the third biopsy. Demethylation was weaker in the progressive upregulated genes, presumably because the overrepresented immunological genes did not change their methylation patterns (Figure 7B).

Hypermethylation was found in downregulated genes. This was more pronounced for the progressive downregulated genes (Figure S5). In CTVT-5 and CTVT-17, the internal and last exons showed progressive hypermethylation. In CTVT-6, the internal exon showed no progressive change in methylation, whereas the last exon showed hypermethylation in the first and second biopsies, which was fainter in the third biopsy (Figure S5). Thus CTVT-6 may be initially epigenetically permissive for changes in gene expression important for regression but this condition is not stable. These results support the hypothesis that epigenetic changes play a significant role in vincristine-induced regression of CTVT. Because most upregulated genes were of host origin, it seems likely that epigenetic changes in stroma and host tissue surrounding the tumor were critical to induce regression.

DISCUSSION

CTVT is unique among naturally transmissible cancers because it can regress (Cohen, 1985; Fassati and Mitchison, 2010). Although spontaneous regression is uncommon, a single dose of vincristine or radiation is often sufficient to cure this cancer in a few weeks (Gonzalez et al., 2000). This suggests that CTVT is particularly susceptible to changes that break tolerance to this cancer; however, a comprehensive analysis of the events leading to regression of natural CTVT was lacking.

To this end we compared tumors that regressed with tumors that did not regress to investigate mechanisms of CTVT regression by transcriptional and methylation profiling. We observed that differential expression of many genes occurred in parallel with changes in the pathology, revealing a stepwise process that begins with a strong inflammatory response and epithelial and keratinocyte proliferation, followed by immune infiltration of T, NK, and B cells, and arrest in the cell cycle. Ultimately, in regressing CTVT, there is loss of tumor cells, cell migration, and tissue remodeling. This process has similarities with wound healing (Strbo et al., 2014), as Mukaratirwa et al. (2004) noted in their histochemical study of CTVT.

The early phase was characterized by a strong and reproducible upregulation of genes involved in epithelial cell and keratinocyte differentiation, including many keratins (*KRT4 KRT13, KRT15, KRT23, KRT78,* and *KRT80*) and epithelia-specific transcription factors such as *TP63* (Mehrazarin et al., 2015; Pellegrini et al., 2001). This suggested that mucosal and/or skin remodeling is one of the first responses characterizing the transition from the P to S phase CTVT. Since keratins were mostly of host origin, we propose that proliferation of keratinocytes and epithelial stem cells of the basal layer is an attempt by the surrounding tissue to contain or replace the malignant tissue. However, these genes were not found in the core signature of regression, suggesting that epithelial cell and keratinocyte differentiation is necessary but not sufficient to trigger regression and may also be associated with the S phase.

Within the early upregulated genes, we detected a significant number of genes involved in inflammation. Expression of genes involved in interferon signaling (*IRF7, ISG15,* and *IFIT1*) was higher in all regressive CTVTs relative to non-regressive CTVTs, suggesting activation of the innate immune response (Schneider et al., 2014). Furthermore, among the most upregulated early genes were chemotactic cytokines *CCL5* and *CCL28* (Sozzani et al., 1996). *CCL5* was statistically significantly upregulated in the regressive CTVTs only; it was one of four early upregulated genes in the core gene signature of regression, and IPA identified CCR5 signaling as one of the most significant canonical pathways consistently detected across regressing CTVTs. CCL5 recruits dendritic cells, monocytes, and lymphocytes (Turner et al., 2014), whereas CCL28, expressed by epithelial cells, is a mucosal-specific cytokine that recruits lymphocytes and eosinophils (Pan et al., 2000).

Keratinocytes can express many chemokines (Sozzani et al., 1996), therefore we propose that their activation and the concomitant tissue inflammation result in further enhanced production of certain chemokines. We propose that, when *CCL5* expression reaches a certain threshold, CTVT regression becomes likely. This is consistent with the notion that, whereas low chronic inflammation has a pro-tumor effect, a dramatic increase in the production of inflammatory mediators by the local host cells induces a switch from chronic to florid inflammation, which triggers infiltration by immune cells (Mantovani et al., 2008). In our case, the initial strong inflammatory response may be induced by vincristine, which causes the release of damage-associated molecular patterns from stressed or dying cells (Kono and Rock, 2008). Because CTVT is an allograft, substantial, chemokine-mediated recruitment of alloreactive T cells into the tumor should induce direct recognition of foreign DLA molecules, triggering acute rejection (Kono and Rock, 2008). In this scenario, low-dose chemotherapy or radiotherapy, far from causing immunosuppression, would in fact elicit inflammation and trigger a cascade of events ultimately leading to CTVT regression.

Thus, our results support the idea of combining low-dose chemotherapy with immune checkpoint therapy to shift the balance toward an acute inflammatory response that may trigger cancer regression in humans (Galluzzi et al., 2015; Mantovani et al., 2008; Minn and Wherry, 2016; Sharma and Allison, 2015). Cancers that have accumulated many mutations, such as CTVT (Murchison et al., 2014), may produce many neo-antigens and be more prone to rejection (Gubin et al., 2015).

Our correlative transcriptome and methylome analysis indicated that most changes in gene expression (up or down) were associated with changes in methylation at particular sites, suggesting that epigenetic mechanisms were at play in eliciting regression. Indeed, epigenetic regulation of MHC-I expression has been reported in the Tasmanian devil facial tumor disease (Siddle et al., 2013). In agreement with previous reports, we found that methylation changes affecting gene expression were clustered around the first exon, internal exons, and the last exon, whereas changes at promoter CpG islands were less frequent (Hovestadt et al., 2014; Li et al., 2010). It is notable that the non-regressing CTVT-6 showed a trend toward re-methylation in the last biopsy, which suggests that the demethylation of upregulated genes needs to be maintained over time for successful regression. Further work is required to understand what causes this re-methylation in CTVT-6. Because most upregulated genes appeared to be of host origin, the methylation analysis points to a critical epigenetic remodeling of host tissue surrounding the tumor and possibly stroma cells.

Previous reports suggested that CTVT was histiocytic on the basis of immunohistochemical detection of lysozyme, α1-antitrypsin and vimentin in about 30%–50% of the tumor cells (Marchal et al., 1997; Mozos et al., 1996). However, our transcriptome analysis did not detect expression changes of these genes at any stage in any of the CTVT samples (although we note that histamine biogenesis was a prominent canonical pathway identified by IPA across all regressive CTVTs). The third biopsy of CTVT-5 demonstrated advanced regression and almost complete loss of the tumor mass. Because we had serial biopsies available, we reasoned that genes whose expression was profoundly downregulated or lost from the second to the third biopsy in CTVT-5 would provide a "signature" of CTVT. Skin cancer and cutaneous melanoma were the most highly significant networks identified by IPA across all CTVTs on the basis of the downregulated genes. Analysis of the core signature of regression also confirmed this result, which was further tested by independently assessing the relative enrichment of this gene signature in the NCI-60 human cancer cell line panel originating from other solid tumors (Pfister et al., 2009). Caution is required though, because our analysis was not performed on isolated CTVT cells and our interpretation rests on the assumption that the particular gene signature was mainly due to the loss of tumor rather than host cells. Despite these limitations, our result seems plausible because melanocytic and non-melanocytic melanoma can form on the genital mucosa (Postow et al., 2012); hence, as it would be suitably accessible for venereal

transmission, melanoma was previously shown to be transplantable across individuals (Scanlon et al., 1965) and it can occasionally regress due to its intrinsic immunogenicity (Papac, 1996). Furthermore, MHC-II expression can be induced in CTVT and melanoma (Johnson et al., 2016; Murgia et al., 2006), where it is associated with an inflammatory signature and a superior response to anti-PD1 antibody therapy (Johnson et al., 2016). Melanoma and the Tasmanian devil's facial tumor are both neural crest-derived cancers (Murchison et al., 2010; Simoes-Costa and Bronner, 2013), which raises the intriguing possibility that some transmissible cancers share a common origin.

In conclusion, our genome-wide scale and systematic analysis of CTVT regression has provided important new information on the interplay between chemotherapy, the host tissue, the host innate and acquired immune system, and the tumor, which may be applicable in understanding regression of human and animal cancers.

STAR★METHODS

Detailed methods are provided in the online version of this paper and include the following:

- KEY RESOURCES TABLE
- CONTACT FOR REAGENT AND RESOURCE SHARING
- EXPERIMENTAL MODEL AND SUBJECT DETAILS
  - Dogs
- METHOD DETAILS
  - RNAseq
  - SNP Mapping
  - Quantitative PCR for Genomic DNA
  - Methylome
- QUANTIFICATION AND STATISTICAL ANALYSIS
  - Analysis of Gene Expression
  - Heatmaps
  - IPA
- DATA AND SOFTWARE AVAILABILITY

SUPPLEMENTAL INFORMATION

Supplemental Information includes five figures and eight tables and can be found with this article online at https://doi.org/10.1016/j.ccell.2018.03.003.

ACKNOWLEDGMENTS

This study was funded by a grant from the UCL Cancer Center Development Fund (to A.F. and S.B.) and the Biotechnology and Biological Sciences Research Council (to A.F. ref. BB/L021404/1). L.M.B. and S.B. were supported by the Wellcome Trust (grant ref. 84071). R.K. and J.K.-V. were supported by the UK Medical Research Council (ref. MC_U12266B). We thank Michael Stratton and Liz Murchison for support with the RNA-seq and helpful discussions, and Chris Monit for bioinformatics support.

AUTHOR CONTRIBUTIONS

G.M. and K.F.d.C. provided the biopsy samples. R.A., H.S., K.F.d.C., L.M.B., and S.B. acquired the data. D.F., L.M.B., H.S., C.V., G.P., J.K.-V., R.K., B.C., R.A.G., R.A.W., S.B., and A.F. analyzed the data. A.F., S.B., G.M., and H.S. designed the experiments. A.F. wrote the paper with input from all co-authors.

DECLARATION OF INTERESTS

The authors declare no competing interests.

Received: March 2, 2017
Revised: December 8, 2017
Accepted: March 1, 2018
Published: April 9, 2018

REFERENCES

Anders, S., and Huber, W. (2010). Differential expression analysis for sequence count data. Genome Biol. *11*, R106.

Anders, S., Pyl, P.T., and Huber, W. (2015). HTSeq–a Python framework to work with high-throughput sequencing data. Bioinformatics *31*, 166–169.

Belov, K. (2012). Contagious cancer: lessons from the devil and the dog. Bioessays *34*, 285–292.

Bird, A.P. (1986). CpG-rich islands and the function of DNA methylation. Nature *321*, 209–213.

Blaine, D.P. (1810). A Domestic Treatise on the Diseases of Horses and Dogs, Fourth Edition (T. Boosey).

Bray, N.L., Pimentel, H., Melsted, P., and Pachter, L. (2016). Near-optimal probabilistic RNA-seq quantification. Nat. Biotechnol *34*, 525–527.

Chiang, H.C., Liao, A.T., Jan, T.R., Wang, Y.S., Lei, H.J., Tsai, M.H., Chen, M.F., Lee, C.Y., Lin, Y.C., Chu, R.M., et al. (2013). Gene-expression profiling to identify genes related to spontaneous tumor regression in a canine cancer model. Vet. Immunol. Immunopathol. *151*, 207–216.

Chu, R.M., Lin, C.Y., Liu, C.C., Yang, S.Y., Hsiao, Y.W., Hung, S.W., Pao, H.N., and Liao, K.W. (2001). Proliferation characteristics of canine transmissible venereal tumor. Anticancer Res. *21*, 4017–4024.

Cohen, D. (1985). The canine transmissible venereal tumor: a unique result of tumor progression. Adv. Cancer Res. *43*, 75–112.

Crum, C.P., and McKeon, F.D. (2010). p63 in epithelial survival, germ cell surveillance, and neoplasia. Annu. Rev. Pathol. *5*, 349–371.

Epstein, R.B., and Bennett, B.T. (1974). Histocompatibility typing and course of canine venereal tumors transplanted into unmodified random dogs. Cancer Res. *34*, 788–793.

Esteller, M. (2005). Aberrant DNA methylation as a cancer-inducing mechanism. Annu. Rev. Pharmacol. Toxicol. *45*, 629–656.

Fassati, A., and Mitchison, N.A. (2010). Testing the theory of immune selection in cancers that break the rules of transplantation. Cancer Immunol. Immunother. *59*, 643–651.

Fujinaga, T., Yamashita, M., Yoshida, M.C., Mizuno, S., Okamoto, Y., Tajima, M., and Otomo, K. (1989). Chromosome analysis of canine transmissible sarcoma cells. Zentralbl. Veterinarmed. A. *36*, 481–489.

Galluzzi, L., Buque, A., Kepp, O., Zitvogel, L., and Kroemer, G. (2015). Immunological effects of conventional chemotherapy and targeted anticancer agents. Cancer Cell *28*, 690–714.

Gentleman, R.C., Carey, V.J., Bates, D.M., Bolstad, B., Dettling, M., Dudoit, S., Ellis, B., Gautier, L., Ge, Y., Gentry, J., et al. (2004). Bioconductor: open software development for computational biology and bioinformatics. Genome Biol. *5*, R80.

Gonzalez, C.M., Griffey, S.M., Naydan, D.K., Flores, E., Cepeda, R., Cattaneo, G., and Madewell, B.R. (2000). Canine transmissible venereal tumour: a morphological and immunohistochemical study of 11 tumours in growth phase and during regression after chemotherapy. J. Comp. Pathol. *122*, 241–248.

Gubin, M.M., Artyomov, M.N., Mardis, E.R., and Schreiber, R.D. (2015). Tumor neoantigens: building a framework for personalized cancer immunotherapy. J. Clin. Invest. *125*, 3413–3421.

Hovestadt, V., Jones, D.T., Picelli, S., Wang, W., Kool, M., Northcott, P.A., Sultan, M., Stachurski, K., Ryzhova, M., Warnatz, H.J., et al. (2014). Decoding the regulatory landscape of medulloblastoma using DNA methylation sequencing. Nature *510*, 537–541.

Hsiao, Y.W., Liao, K.W., Hung, S.W., and Chu, R.M. (2004). Tumor-infiltrating lymphocyte secretion of IL-6 antagonizes tumor-derived TGF-beta 1 and restores the lymphokine-activated killing activity. J. Immunol. *172*, 1508–1514.

Idowu, L. (1977). The chromosomes of the transmissible venereal tumour of dogs in Ibadan, Nigeria. Res. Vet. Sci. *22*, 271–273.

Johnson, D.B., Estrada, M.V., Salgado, R., Sanchez, V., Doxie, D.B., Opalenik, S.R., Vilgelm, A.E., Feld, E., Johnson, A.S., Greenplate, A.R., et al. (2016). Melanoma-specific MHC-II expression represents a tumour-autonomous phenotype and predicts response to anti-PD-1/PD-L1 therapy. Nat. Commun. *7*, 10582.

Kaetzel, C.S. (2005). The polymeric immunoglobulin receptor: bridging innate and adaptive immune responses at mucosal surfaces. Immunol. Rev. *206*, 83–99.

Katzir, N., Rechavi, G., Cohen, J.B., Unger, T., Simoni, F., Segal, S., Cohen, D., and Givol, D. (1985). "Retroposon" insertion into the cellular oncogene c-myc in canine transmissible venereal tumor. Proc. Natl. Acad. Sci. USA *82*, 1054–1058.

Kono, H., and Rock, K.L. (2008). How dying cells alert the immune system to danger. Nat. Rev. Immunol. *8*, 279–289.

Leclerc-Mercier, S., Bodemer, C., Furio, L., Hadj-Rabia, S., de Peufeilhoux, L., Weibel, L., Bursztejn, A.C., Bourrat, E., Ortonne, N., Molina, T.J., et al. (2016). Skin biopsy in Netherton syndrome: a histological review of a large series and new findings. Am. J. Dermatopathol. *38*, 83–91.

Li, H., Handsaker, B., Wysoker, A., Fennell, T., Ruan, J., Homer, N., Marth, G., Abecasis, G., and Durbin, R. (2009). The sequence alignment/map format and SAMtools. Bioinformatics *25*, 2078–2079.

Li, J., Ireland, G.W., Farthing, P.M., and Thornhill, M.H. (1996). Epidermal and oral keratinocytes are induced to produce RANTES and IL-8 by cytokine stimulation. J. Invest. Dermatol. *106*, 661–666.

Li, Y., Zhu, J., Tian, G., Li, N., Li, Q., Ye, M., Zheng, H., Yu, J., Wu, H., Sun, J., et al. (2010). The DNA methylome of human peripheral blood mononuclear cells. PLoS Biol. *8*, e1000533.

Love, M.I., Huber, W., and Anders, S. (2014). Moderated estimation of fold change and dispersion for RNA-seq data with DESeq2. Genome Biol. *15*, 550.

Mantovani, A., Romero, P., Palucka, A.K., and Marincola, F.M. (2008). Tumour immunity: effector response to tumour and role of the microenvironment. Lancet *371*, 771–783.

Marchal, T., Chabanne, L., Kaplanski, C., Rigal, D., and Magnol, J.P. (1997). Immunophenotype of the canine transmissible venereal tumour. Vet. Immunol. Immunopathol. *57*, 1–11.

Mehrazarin, S., Chen, W., Oh, J.E., Liu, Z.X., Kang, K.L., Yi, J.K., Kim, R.H., Shin, K.H., Park, N.H., and Kang, M.K. (2015). The p63 gene is regulated by grainyhead-like 2 (GRHL2) through reciprocal feedback and determines the epithelial phenotype in human keratinocytes. J. Biol. Chem. *290*, 19999–20008.

Metzger, M.J., Reinisch, C., Sherry, J., and Goff, S.P. (2015). Horizontal transmission of clonal cancer cells causes leukemia in soft-shell clams. Cell *161*, 255–263.

Metzger, M.J., Villalba, A., Carballal, M.J., Iglesias, D., Sherry, J., Reinisch, C., Muttray, A.F., Baldwin, S.A., and Goff, S.P. (2016). Widespread transmission of independent cancer lineages within multiple bivalve species. Nature *534*, 705–709.

Minn, A.J., and Wherry, E.J. (2016). Combination cancer therapies with immune checkpoint blockade: convergence on interferon signaling. Cell *165*, 272–275.

Moll, R., Divo, M., and Langbein, L. (2008). The human keratins: biology and pathology. Histochem. Cell Biol. *129*, 705–733.

Mozos, E., Mendez, A., Gomez-Villamandos, J.C., Martin De Las Mulas, J., and Perez, J. (1996). Immunohistochemical characterization of canine transmissible venereal tumor. Vet. Pathol. *33*, 257–263.

Mukaratirwa, S., Chimonyo, M., Obwolo, M., Gruys, E., and Nederbragt, H. (2004). Stromal cells and extracellular matrix components in spontaneous canine transmissible venereal tumour at different stages of growth. Histol. Histopathol. *19*, 1117–1123.

Murchison, E.P. (2008). Clonally transmissible cancers in dogs and Tasmanian devils. Oncogene *27* (*Suppl 2*), S19–S30.

Murchison, E.P., Tovar, C., Hsu, A., Bender, H.S., Kheradpour, P., Rebbeck, C.A., Obendorf, D., Conlan, C., Bahlo, M., Blizzard, C.A., et al. (2010). The Tasmanian devil transcriptome reveals Schwann cell origins of a clonally transmissible cancer. Science *327*, 84–87.

Murchison, E.P., Wedge, D.C., Alexandrov, L.B., Fu, B., Martincorena, I., Ning, Z., Tubio, J.M., Werner, E.I., Allen, J., De Nardi, A.B., et al. (2014). Transmissible [corrected] dog cancer genome reveals the origin and history of an ancient cell lineage. Science *343*, 437–440.

Murgia, C., Pritchard, J.K., Kim, S.Y., Fassati, A., and Weiss, R.A. (2006). Clonal origin and evolution of a transmissible cancer. Cell *126*, 477–487.

Ngan, V.K., Bellman, K., Hill, B.T., Wilson, L., and Jordan, M.A. (2001). Mechanism of mitotic block and inhibition of cell proliferation by the semisynthetic *Vinca* alkaloids vinorelbine and its newer derivative vinflunine. Mol. Pharmacol. *60*, 225–232.

Novinski, M. (1876). Zur Frage uber die Impfung der krebsigen Geschwulste. Zentralbl. Med. Wissensch. *14*, 790–791.

Pan, J., Kunkel, E.J., Gosslar, U., Lazarus, N., Langdon, P., Broadwell, K., Vierra, M.A., Genovese, M.C., Butcher, E.C., and Soler, D. (2000). A novel chemokine ligand for CCR10 and CCR3 expressed by epithelial cells in mucosal tissues. J. Immunol. *165*, 2943–2949.

Papac, R.J. (1996). Spontaneous regression of cancer. Cancer Treat. Rev. *22*, 395–423.

Pellegrini, G., Dellambra, E., Golisano, O., Martinelli, E., Fantozzi, I., Bondanza, S., Ponzin, D., McKeon, F., and De Luca, M. (2001). p63 identifies keratinocyte stem cells. Proc. Natl. Acad. Sci. USA *98*, 3156–3161.

Perez, J., Day, M.J., and Mozos, E. (1998). Immunohistochemical study of the local inflammatory infiltrate in spontaneous canine transmissible venereal tumour at different stages of growth. Vet. Immunol. Immunopathol. *64*, 133–147.

Pfister, T.D., Reinhold, W.C., Agama, K., Gupta, S., Khin, S.A., Kinders, R.J., Parchment, R.E., Tomaszewski, J.E., Doroshow, J.H., and Pommier, Y. (2009). Topoisomerase I levels in the NCI-60 cancer cell line panel determined by validated ELISA and microarray analysis and correlation with indenoisoquinoline sensitivity. Mol. Cancer Ther. *8*, 1878–1884.

Postow, M.A., Hamid, O., and Carvajal, R.D. (2012). Mucosal melanoma: pathogenesis, clinical behavior, and management. Curr. Oncol. Rep. *14*, 441–448.

Proost, P., Wuyts, A., and van Damme, J. (1996). The role of chemokines in inflammation. Int. J. Clin. Lab. Res. *26*, 211–223.

Rebbeck, C.A., Thomas, R., Breen, M., Leroi, A.M., and Burt, A. (2009). Origins and evolution of a transmissible cancer. Evolution *63*, 2340–2349.

Robinson, J.T., Thorvaldsdottir, H., Winckler, W., Guttman, M., Lander, E.S., Getz, G., and Mesirov, J.P. (2011). Integrative genomics viewer. Nat. Biotechnol. *29*, 24–26.

Sarwal, M., Chua, M.S., Kambham, N., Hsieh, S.C., Satterwhite, T., Masek, M., and Salvatierra, O., Jr. (2003). Molecular heterogeneity in acute renal allograft rejection identified by DNA microarray profiling. N. Engl. J. Med. *349*, 125–138.

Scanlon, E.F., Hawkins, R.A., Fox, W.W., and Smith, W.S. (1965). Fatal homotransplanted melanoma: a case report. Cancer *18*, 782–789.

Schiffman, J.D., and Breen, M. (2015). Comparative oncology: what dogs and other species can teach us about humans with cancer. Philos. Trans. R. Soc. Lond. B Biol. Sci. *370*, https://doi.org/10.1098/rstb.2014.0231.

Schlosser, A., Pilecki, B., Hemstra, L.E., Kejling, K., Kristmannsdottir, G.B., Wulf-Johansson, H., Moeller, J.B., Fuchtbauer, E.M., Nielsen, O., Kirketerp-Moller, K., et al. (2016). MFAP4 promotes vascular smooth muscle migration, proliferation and accelerates neointima formation. Arterioscler. Thromb. Vasc. Biol. *36*, 122–133.

Schneider, W.M., Chevillotte, M.D., and Rice, C.M. (2014). Interferon-stimulated genes: a complex web of host defenses. Annu. Rev. Immunol. *32*, 513–545.

Sharma, P., and Allison, J.P. (2015). The future of immune checkpoint therapy. Science *348*, 56–61.

Siddle, H.V., Kreiss, A., Tovar, C., Yuen, C.K., Cheng, Y., Belov, K., Swift, K., Pearse, A.M., Hamede, R., Jones, M.E., et al. (2013). Reversible epigenetic down-regulation of MHC molecules by devil facial tumour disease illustrates immune escape by a contagious cancer. Proc. Natl. Acad. Sci. USA *110*, 5103–5108.

Simoes-Costa, M., and Bronner, M.E. (2013). Insights into neural crest development and evolution from genomic analysis. Genome Res. *23*, 1069–1080.

Soneson, C., Love, M.I., and Robinson, M.D. (2015). Differential analyses for RNA-seq: transcript-level estimates improve gene-level inferences. F1000Res. *4*, 1521.

Sozzani, S., Locati, M., Allavena, P., Van Damme, J., and Mantovani, A. (1996). Chemokines: a superfamily of chemotactic cytokines. Int. J. Clin. Lab. Res. *26*, 69–82.

Stramer, B., and Martin, P. (2005). Cell biology: master regulators of sealing and healing. Curr. Biol. *15*, R425–R427.

Strbo, N., Yin, N., and Stojadinovic, O. (2014). Innate and adaptive immune responses in wound epithelialization. Adv. Wound Care (New Rochelle) *3*, 492–501.

Taiwo, O., Wilson, G.A., Morris, T., Seisenberger, S., Reik, W., Pearce, D., Beck, S., and Butcher, L.M. (2012). Methylome analysis using MeDIP-seq with low DNA concentrations. Nat. Protoc. *7*, 617–636.

Thrall, D.E. (1982). Orthovoltage radiotherapy of canine transmissible venereal tumors. Vet. Radiol. *23*, 217–219.

Trapnell, C., Pachter, L., and Salzberg, S.L. (2009). TopHat: discovering splice junctions with RNA-Seq. Bioinformatics *25*, 1105–1111.

Turner, M.D., Nedjai, B., Hurst, T., and Pennington, D.J. (2014). Cytokines and chemokines: at the crossroads of cell signalling and inflammatory disease. Biochim. Biophys. Acta *1843*, 2563–2582.

Vogl, T., Tenbrock, K., Ludwig, S., Leukert, N., Ehrhardt, C., van Zoelen, M.A., Nacken, W., Foell, D., van der Poll, T., Sorg, C., et al. (2007). Mrp8 and Mrp14 are endogenous activators of Toll-like receptor 4, promoting lethal, endotoxin-induced shock. Nat. Med. *13*, 1042–1049.

Wilson, G.A., Dhami, P., Feber, A., Cortazar, D., Suzuki, Y., Schulz, R., Schar, P., and Beck, S. (2012). Resources for methylome analysis suitable for gene knockout studies of potential epigenome modifiers. Gigascience *1*, 3.

Yang, T.J., and Jones, J.B. (1973). Canine transmissible venereal sarcoma: transplantation studies in neonatal and adult dogs. J. Natl. Cancer Inst. *51*, 1915–1918.

STAR★METHODS

KEY RESOURCES TABLE

REAGENT or RESOURCE	SOURCE	IDENTIFIER
Antibodies		
anti-5-methylcytosine antibody	Diagenode	C15200006-100
Biological Samples		
CTVT biopsies (see STAR Methods for details)	Department of Veterinary Sciences, University of Messina, Italy and Veterinary Hospital "Dr Halim Atique" - Centro Universitário de Rio Preto (UNIRP), São José do Rio Preto, São Paulo, Brazil	N/A
Chemicals, Peptides, and Recombinant Proteins		
Vincristine (Italy)<br>Vincristine (Brazil)	Teva Italia<br>Libbs	038549010<br>7896094202870
Lidocaine	Zoetis	100319019
Proteinase K (ChIP grade)	Diagenode	C06050002
Critical Commercial Assays		
SYBR® Green Mix	LifeTechnologies/Thermo Fisher	4309155
DNAse I kit	Thermo Fisher	AM1906
High-Capacity cDNA Reverse Transcription Kit	Applied Biosystems/Thermo Fisher	4368814
QuantiTect SYBR Green RT-PCR Kit	Qiagen	204243
AmpureXT magnetic beads	AutoQ Biosciences	AQ 60050
Illumina TruSeq RNA Library Prep kit v2	Illumina	RS-122-2001
KAPA Stranded mRNA-Seq Kit	KAPA Bioscience	KK8420
Auto-MeDIP kit	Diagenode	AF-Auto01-0016
End repair module	New England Biolabs	E6050L
A-tail module	New England Biolabs	E6053L
Adapter ligation module	New England Biolabs	E6056L
MESA BLUE qPCR MasterMix Plus	Eurogentec	RT-SY2X-03+WOUB
High-fidelity Phusion polymerase (5×)	New England Biolabs	M0530L
dNTP mix	New England Biolabs	N0447L
Deposited Data		
RNAseq for CTVT-5 and CTVT-6	This paper	ArrayExpress E-MTAB-5488
RNAseq for 7xx CTVTs	This paper	ArrayExpress E-MTAB-5889
MeD-IP	This paper	ArrayExpress E-MTAB-5495
CTVT genome 1	Murchison et al., 2014	European Nucleotide Archive SAMEA2358413
CTVT genome 2	Murchison et al., 2014	European Nucleotide Archive SAMEA2358415
Cancer cells transcriptomes	ArrayExpress	E-GEOD-32474
Oligonucleotides		
β-ACTIN Forward: CTCCATCATGAAGTGTGACGTTG	Murgia et al., 2006	N/A
β-ACTIN Reverse: CGATGATCTTGATCTTCATTGTGC	Murgia et al., 2006	N/A

(*Continued on next page*)

Continued

REAGENT or RESOURCE	SOURCE	IDENTIFIER
DLA DQA-1 tumour. Forward: GAATTTGATGGCGATGAGTT	Murgia et al., 2006	N/A
DLA DQA-1 tumour Reverse: TCAGGATGTTCAAGTTTTGT TTTAT	Murgia et al., 2006	N/A
DLA DQA-1 common. Forward ACTACGGCATAAATGTCTA CCAGTC	Murgia et al., 2006	N/A
DLA DQA-1 common Reverse: CAAGTTTCTCAGTGCAC CCTGT	Murgia et al., 2006	N/A
LINE-MYC Forward: AGGGTTTCCCATCCTTTAACATT	Murgia et al., 2006	N/A
LINE-MYC Reverse: AGATAAGAAGCTTTTGCACAGCAA	Murgia et al., 2006	N/A
PE.Adapter.1.0: ACACTCTTTCCCTACACGACGCTCTTCCGATC*T (*) = phosphothiolate modification	This Paper	N/A
PE.Adapter.2.0: [Phos]GATCGGAAGAGCGGTTCAGCA GGAATGCCGAG [Phos] = 3′phosphate group	This Paper	N/A
Methylated qPCR Forward: GGTGAACTTCCGATAGTG	This Paper	N/A
Methylated qPCR Reverse: CAGTCATAGATGGTCGGT	This Paper	N/A
Unmethylated Forward: GTTAGAGCCTGCATAACG	This Paper	N/A
Unmethylated Reverse: GAAAGAGCACTGGCTAAC	This Paper	N/A
PCR_primer_PE_1.0: AATGATACGGCGACCACCGAGATCTACACTCTTTCCCT ACACGACGCTCTTCCGATC*T; (*) = phosphothiolate modification	This Paper	N/A
PCR_primer_PE_2.0: CAAGCAGAAGACGGCATACGAGA TCGGTCTCGGCATTCCTGCTGAACCGCTCTTCCGATC*T [Phos] = 3′phosphate group	This Paper	N/A
**Software and Algorithms**		
Ingenuity Pathway Analysis (IPA®)	Qiagen	www.qiagen.com/ingenuity
MEV software suite	TM4 MeV	http://www.tm4.org/mev.html
PicardTools	Broad Institute	http://broadinstitute.github.io/picard/
HTSeq-count	Anders et al., 2015	http://www-huber.embl.de/users/anders/HTSeq
TopHat v2.0.13	Trapnell et al., 2009	https://ccb.jhu.edu/software/tophat/index.shtml
R/BioConductor	Gentleman et al., 2004	https://www.bioconductor.org/
MeDUSA	Wilson et al., 2012	https://www.ucl.ac.uk/cancer/research/department-cancer-biology/medical-genomics-group/past-projects/medusa-project/medusa
DESeq BioConductor library	Anders and Huber, 2010	https://bioconductor.org/packages/release/bioc/html/DESeq.html
DESeq2 BioConductor library	Love et al., 2014.	http://bioconductor.org/packages/release/bioc/html/DESeq2.html

CONTACT FOR REAGENT AND RESOURCE SHARING

Further information and requests for resources and reagents should be directed to and will be fulfilled by the Lead Contact Ariberto Fassati (a.fassati@ucl.ac.uk).

EXPERIMENTAL MODEL AND SUBJECT DETAILS

Dogs

CTVT biopsies were collected from 9 dogs as detailed below:

Sample	Age of Dog (Years)	Sex	Breed	Location	Year of Collection	Site of Tumor	Treatment
CTVT-5	3	M	Mixed	Messina (Italy)	2009	Penis	Vincristine 2 sessions
CTVT-6	4	F	Mixed	Messina (Italy)	2009	Vestibulum	Vincristine 2 sessions
CTVT-17	5	M	Mixed	Reggio Calabria (Italy)	2009	Penis	Vincristine 2 sessions
CTVT-761	5	F	Mixed	São José do Rio Preto (Brazil)	2012	Vagina	Vincristine 6 sessions
CTVT-765	4	F	Border-Collie	São José do Rio Preto (Brazil)	2012	Vulva	Vincristine 7 sessions
CTVT-766	7	F	Shih Tzu	São José do Rio Preto (Brazil)	2012	Vagina	Vincristine 4 sessions
CTVT-772	5	M	Mixed	São José do Rio Preto (Brazil)	2013	Penis	Vincristine 6 sessions
CTVT-774	4	M	Mixed	São José do Rio Preto (Brazil)	2013	Nose	Vincristine 8 sessions
CTVT-775	3	M	Border-Collie	São José do Rio Preto (Brazil)	2013	Penis	Vincristine 7 sessions

Biopsies of CTVT-5 and CTVT-6 were collected from dogs undergoing diagnostic assessment at the Department of Veterinary Sciences, University of Messina, Italy. Biopsy of CTVT-17 was collected at a kennel in Reggio Calabria, Italy. Biopsies of CTVT-761, 765, 766, 772, 774 and 775 were collected at the Veterinary Hospital ''Dr Halim Atique'' - Centro Universitário de Rio Preto (UNIRP), São José do Rio Preto, São Paulo, Brazil. All the procedures on animals treated in Italy were in agreement with welfare and ethics European directives and were approved by the University of Messina, under protocol number ORME07PFLB. All the procedures carried out in Brazil were approved by the Animal Research Ethics Committee of the São Paulo State University at Jaboticabal, São Paulo, Brazil, under protocol number 24674/2012. All dogs were in good health at the time of the consultation. Diagnosis of CTVT was done by means of visual inspection of the tumor and evaluation of size, morphology and location, followed by cytological smears, stained with May-Grünwald Giemsa. Apoptotic cells and infiltrating cells were counted manually. Biopsies were collected by surgical excision, after local infiltrations of lidocaine, frozen in liquid N_2 and processed for histology and immunohistochemistry. Dogs with confirmed CTVT were treated as follows: CTVT-5 and 6, vincristine sulfate 0.025 mg/Kg iv once and biopsies were collected at day 0 (before treatment), day 6 and day 14 after treatment; CTVT-17: 0.025 mg/Kg iv vincristine sulfate once and biopsies collected at day 0, 22, and 48 after treatment; CTVT-761, 765, 766, 772, 774, 775, vincristine sulfate 0.5 mg/m^2 iv at weekly intervals and biopsies collected at day 0 (before treatment) and at day 28 after the third dose of vincristine.

METHOD DETAILS

RNAseq

Nucleic acids were extracted from biopsy 1 (pre-therapy), biopsy 2 (day 6 after vincristine) and biopsy 3 (day 14 after vincristine) of CTVT-5 and CTVT-6; from biopsy 1 (pre-therapy), biopsy 2 (day 22 after vincristine) and biopsy 3 (day 48 after vincristine) of CTVT 17 and from biopsy 1 (pre-therapy) and biopsy 2 (day 28 - after the third dose of vincristine) of CTVT-761, 765, 766, 772, 774, 775 using Qiagen RNAeasy Blood & Tissue kit following the manufacturer's instructions.

For CTVT-5, CTVT-6 and CTVT-17, 250 ng of total RNA was prepared for sequencing following the Illumina TruSeq mRNA (unstranded) protocol with insert size 150-250, multiplexed, and sequenced (V2 High Output kit, 54bp PE) on an Illumina Nextseq to yield an average of > 15 million reads per sample. Data was de-multiplexed using bcl2fastq v 2.16. Read QC reports were generated in Illumina Basespace. FASTQ files passing read QC were analyzed on the WTSI genomics cluster using an in house pipeline (Python/R). Sequencing reads were aligned to Canfam 2.0 reference genome using TopHat v2.0.13 (Trapnell et al., 2009); GTF files describing genes features were obtained from the Ensembl website (http://www.ensembl.org/info/data/ftp/index.html). Duplicate reads were then removed using PicardTools (http://broadinstitute.github.io/picard/) /) and read counts per gene generated using HTSeq-count (http://www-huber.embl.de/users/anders/HTSeq).

For CTVT-761, 765, 766, 772, 774, 775, 1 μg of total RNA was prepared for sequencing following the KAPA Stranded mRNA-Seq Kit (KAPA Bioscience) protocol with insert size 150-250, multiplexed, and sequenced (V2 High Output kit, 54bp PE) on an Illumina Nextseq to yield an average of > 10 million reads per sample. RNAseq analysis, data were de-multiplexed using bcl2fastq v 2.17. Paired end reads were mapped to dog transcriptome reference sequence Canfam 2.0 to make it directly comparable with CTVT 5 and 6. Mapping and generation of read counts per gene were done using Kallisto (Bray et al., 2016).

SNP Mapping

Integrative Genomic Viewer (IGV) was used to visualize mapped reads from CTVT samples and two published CTVTs (Accession numbers: SAMEA2358413 and SAMEA2358415, downloaded from the ENA) and to manually inspect SNPs (Robinson et al., 2011).

Quantitative PCR for Genomic DNA

qPCR to detect CTVT and host DNA in CTVT-761, 765, 766, 772, 774 and 775 was performed using an Applied Biosystems 7900HT Fast Real-Time PCR system in a final volume of 20 μL using SYBR® Green Mix (Life Technologies), 20 ng genomic DNA and 5 μM primers as described in the Key Resources Table. Cycling parameters were 95°C, 10 min for 1 cycle followed by 95°C, 15 s and 60°C, 60 s for 40 cycles. Standard curves for *LINE-MYC*, *ACTB*, *DLA DQA-1* tumor and *DLA DQA-1* common were generated using a CTVT sample. Relative *LINE-MYC*, *ACTB*, *DLA DQA-1* (tumor) and *DLA DQA-1* (common) amplification values were estimated for each sample using the standard curve. Each qPCR was performed in triplicate. Values were normalized against a standard curve that was generated for each primer set using CTVT tumour 29T sample as reference, which was previously characterized (Murchison et al., 2014). This linear standard curve was generated from a range of known relative DNA concentrations of 29T, which could be used to calibrate each qPCR reaction and calculate the relative amount of DNA input. Relative DNA input for *LINE-MYC, DLA DQA-1* tumor and *DLA DQA-1* common was then normalized to *ACTB*. The relative standard curve was generated according to the Guide to performing relative quantitation of gene expression using real-time quantitative PCR – Applied Biosystems (Carlsbad, California, USA).

Methylome

Methylated DNA Immunoprecipitation (MeDIP)-seq was performed using 600 ng genomic DNA from biopsies 1, 2 and 3 of CTVT-5, CTVT-6 and CTVT-17. DNA was sonicated on a Diagenode Bioruptor (4 x 15 min cycles set to high intensity) to produce a median fragment length of 180–230 and verified using a 2100 Bioanalyzer with DNA1000 chips (Agilent; p/n 5067-1504). Sample DNA was prepared for next-generation sequencing as follows:

Firstly, DNA was end repaired: reagents (NEBNext End Repair Enzyme mix: 5 μl; NEBNext End Repair Reaction Buffer (10X): 10 μl; Fragmented DNA: 85 μl) were mixed on ice in a sterile PCR tube and incubated in a thermocycler at 20°C for 30 min. The reaction was placed on ice after incubation then spun briefly in a microfuge for ~10 s to collect condensate. Next the reaction was purified with Ampure XP purification beads: end-repaired DNA and 1.8× volumes of magnetic beads were added to a clean 1.5 ml microcentrifuge tube, pipette mixed to homogenize and allow DNA to bind to magnetic beads. The mixture was incubated at room temperature for 5 min then placed on a magnetic rack to collect beads/DNA. Supernatant was aspirated off and discarded. Next, 200 μl 70% ethanol was added to the beads and left for 30 s. Supernatant was aspirated off and discarded. Repeat ethanol wash. Leaving the samples in magnetic rack, the magnetic rack was placed in oven for 10-15 min to evaporate residual ethanol. When beads looked drab, the sample was taken off the magnetic rack and 37 μl Tris-HCl was added to liberate DNA from beads. The mixture was gently pipette mixed and incubated for 1 min. The sample was placed back on the magnetic rack to separate the beads. Supernatant was aspirated off and carried to the dA- Tailing step. Following purification, DNA was dA- tailed: reagents (Klenow Fragment (3'→5' exo-): 3 μl; NEB Next dA-tailing Reaction Buffer (10X): 5 μl; water: 5 μl; purified DNA: 37 μl) were mixed on ice in a sterile PCR tube and incubated in a thermal cycler at 37°C for 30 min. Following incubation the samples were purified with Ampure XP purification beads and eluted in 25 μl Tris-HCl for the next step.

Next, paired-end sequencing adapters were added: reagents (Quick T4 DNA Ligase:5 μl; Quick Ligation Reaction Buffer (5x): 10 μl; paired-end sequencing adapters [PE.Adapter.1.0 and PE.Adapter.2.0]: 10 μl; purified DNA: 25 μl) were mixed on ice in a sterile PCR tube and incubated in a thermocycler at 18°C for 15 min. Following incubation the samples were purified with Ampure XP purification beads and eluted in 25 μl Tris-HCl for the next step.

Following purification of adapter-ligated samples, each sample was spiked with approximately $5x10^{-7}$ pmoles methylated/unmethylated Enterobacteria phage λ DNA derived by PCR of select regions described elsewhere (Taiwo et al., 2012); methylated fragments were obtained by *in vitro* methylation using *SssI* methyltransferase. Ten percent sample DNA was reserved as input for quality control purposes (see below). The remaining DNA was incubated with 150 ng anti-5-methylcytidine at 4°C for 15 hrs using automation and manufacturer reagents and protocols (Diagenode). Following immunoprecipitation, DNA was incubated with 1 μg proteinase K (Diagenode) at 55°C for 15 min then 95°C for 15 min. Each sample was subject to quality control using triplicate quantitative PCR (qPCR) reactions with primers designed to amplify methylated/unmethylated control DNA. Reagents (Eurogentec MESABlue qPCR master mix (2×): 6.25 μl; Methylated/Unmethylated Forward and Reverse primer pairs (10 μM): 0.625 μl; water: 4.375 μl; MeDIP or input DNA: 1.25 μl) were mixed on ice and incubated on an ABI 7900 qPCR machine under the following conditions: 95°C for 60 s ; 40 cycles of 95°C for 30 s and 60°C for 60 s then 72°C for 5 min. After confirming enrichment of methylated DNA in the immunoprecipitated fraction (see Taiwo et al., 2012), DNA was purified using AmpureXP magnetic beads

Following purification, immunoprecipitated DNA samples enriched by adapter-mediated PCR: reagents (water: 2.5 μl; 5x Phusion buffer: 10 μl; NEB dNTPs: 1.5 μl; PCR_PE_1.0 (10 μM stock): 5 μl; PCR_PE_ 2.0 (10 μM stock): 5 μl; purified DNA: 25 μl; Phusion Polymerase: 1 μl) were mixed on ice in a sterile PCR tube and incubated under the following conditions: 95°C for 30 s; 12 cycles 98°C for 20 s, 65°C for 30 s, 72°C for 30 s, then 72°C for 5 min. Following incubation the samples were purified with Ampure XP purification beads and eluted in 15 μl Tris-HCl. Next, DNA sequencing libraries were size-selected: samples were mixed with 5x loading dye and run out on a 2% TBE agarose gel containing EtBr (1.0 μg/ml), maintaining a minimum of 2 cm between wells. Electrophoresis was performed at 100 volts for 100 min. Following gel electrophoresis, the gel was transferred to a UV transilluminator with a strip of aluminum foil beneath sample wells. Using a clean scalpel, a 300-350 bp slice was excided and transferred to a clean 1.5 ml microcentrifuge tube.

Following gel-excision, samples were purified using QIAGEN Gel Extraction kits: 3 volumes of buffer QG were added to 1 volume gel and vortexed to mix. QG-gel mix was incubated at room temperature until fully dissolved. Next, 10 μl 3 M sodium acetate (pH 5.0)

was added and mixed. Next, 1 gel volume of isopropanol was added to the sample and mixed by inversion. The sample was transferred to a MinElute column and centrifuged at 16,000 rpm for 1 min. The flow through was discarded and 500 μl buffer QG was added to the empty sample tube and mixed on a vortex. Next, the 500 μl buffer QG was transferred to MinElute column from the previous step; left to stand for 1 min and centrifuged at max speed for 1 min. The flow through was discarded. Using a pipette, any residual buffer QG from inside the MinElute column was aspirated and discard before washing. To wash, 750 μl PE wash buffer were added to the clean-up column. The column was gently inverted several times to thoroughly wash and centrifuged at max for 1 min. The flow through was discarded and the clean-up column placed back in the same tube. The column was centrifuged for an additional 2 min and visually inspected to ensure that there was no residual solution in the column. The column reservoir was placed in a clean 1.5 ml microcentrifuge tube and DNA eluted by adding 10 μl EB directly onto column the membrane, left to stand for 5 min then centrifuged at max speed for 1 min. 1 μl size selected DNA was assessed on an Agilent Bioanalyzer using DNA High Sensitivity Chips to determine concentration and molarity.

Samples were sequenced on an Illumina GAIIx with 36bp paired-end reads. Downstream bioinformatic processing of reads prior to analysis (i.e., sequence quality control, alignment and filtering) was performed with the MeDUSA pipeline (Wilson et al., 2012). To construct the methylation boxplots, chromosomal coordinates were determined for the first exon, the first intron, internal exons, internal introns, and the final exon for each gene, in addition to coordinates corresponding to 2 kb regions up- and downstream of the first and final exons respectively. These coordinates were used to construct 20 equally spaced bins for each such feature per gene. Bam files for each sample were converted into individual wig files (Li et al., 2009), from which mean levels of methylation were calculated for each bin. These values were then concatenated to investigate larger-scale methylation patterns across gene sets of interest (e.g. non-expressed genes, S/P "early" up-regulated genes, etc.) using in-house Perl scripts and R.

QUANTIFICATION AND STATISTICAL ANALYSIS

Analysis of Gene Expression

For CTVT-5 and CTVT-6, R/BioConductor (Gentleman et al., 2004) was used to import the mapped count data and the DESeq library (Anders and Huber, 2010) was used to normalize the data, estimate variance, filter low expression genes and then predict differentially expressed genes. Specifically, a filtering step was applied to remove low expression genes whose sum of counts across all conditions was within the lowest 40% quantile (9886/24660 genes). Counts for the 14774 remaining genes were then fitted to a negative binomial generalized linear model using a multi-factorial design matrix (tumor stage, dog) and applying a fold-change cut-off of +/- 10 and a Benjamini-Hochberg adjusted p value cut-off of 0.01.

For the 7xx CTVTs, Tximport was used to import the mapped counts data into R and summarize the transcripts-level data into gene level as described ((Soneson et al., 2015). Counts for 21047 genes were normalized and further analyzed using DESeq2 and the SARTools packages (Love et al., 2014). Differential gene expression was performed by fitting counts to a negative binomial generalized linear model using a multi-factorial design matrix (design formula of: *regression* + *regression:dog* + *regression:time*).

Heatmaps

The 1016 genes that passed the cut-off (FC>10, adj-p< 0.01) in the old CTVTs and the 1350 genes that passed the same cut-off in the 7xx CTVTs were included in the heatmaps. To generate heatmaps that are consistent across samples, for panel A we calculated log 2 differential expression of the 1016 genes relative to the geometric mean of CTVT-5 B1 and CTVT-6 B1, which corresponds to the mean of the Log2FC. Genes were sorted by relative differential expression of B3. For panel B, we similarly calculated differential expression of the 1350 genes relative to the geometric mean of the five B1 samples. Genes were then sorted by the geometric average relative expression increase in B2s from the three regressing dogs. The raw data used to generate the heatmaps are provided in Table S2. Heatmaps were generated using heatmap.2 function in the package gplots in R (Gregory R. Warnes, Ben Bolker, Lodewijk Bonebakker, Robert Gentleman, Wolfgang Huber Andy Liaw, Thomas Lumley, Martin Maechler, Arni Magnusson, Steffen Moeller, Marc Schwartz and Bill Venables (2016). gplots: Various R Programming Tools for Plotting Data. R package version 3.0.1. https://CRAN.R-project.org/package=gplots).

IPA

The networks and functional analyses were generated through the use of QIAGEN's Ingenuity Pathway Analysis (IPA®, QIAGEN Redwood City, www.qiagen.com/ingenuity). Genes that reached a cut-off of FC>10 and an adj-p< 0.01 were included in the IPA analysis. Comparative analyses were performed using the specific IPA function, selecting individual IPA outputs and clustering pathways based on both statistical significance (Fisher's exact test) and similarity. Diagrams were generated using the Pathdesigner function from the most highly significant and represented networks in the Diseases and Bio-Functions and Canonical Pathways.

DATA AND SOFTWARE AVAILABILITY

The accession number for the RNAseq of CTVT-5 and CTVT-6 reported in this paper is ArrayExpress: E-MTAB-5488.

The accession number for the RNAseq of 7xx CTVTs (761, 765, 766, 772, 774 and 775) reported in this paper is ArrayExpress: E-MTAB-5889.

The accession number for the MeDIP for CTVT-5, 6 and 17 reported in this paper is ArrayExpress: E-MTAB-5495.

The accession number for the CTVT genome number 1 reported in (Murchison et al., 2014) is European Nucleotide Archive: SAMEA2358413. The accession number for CTVT genome number 2 reported in (Murchison et al., 2014) is European Nucleotide Archive SAMEA2358415.

The accession number for the cancer cells transcriptome reported in this paper is ArrayExpress: E-GEOD-32474.

MeDUSA software: https://www.ucl.ac.uk/cancer/research/department-cancer-biology/medical-genomics-group/past-projects/medusa-project/medusa.

CRAN software: https://CRAN.R.

DESeq BioConductor library: https://bioconductor.org/packages/release/bioc/html/DESeq.html.

DESeq2 BioConductor library: http://bioconductor.org/packages/release/bioc/html/DESeq2.html.

Cancer Cell, Volume *33*

Supplemental Information

Molecular Signatures of Regression of the Canine Transmissible Venereal Tumor

Dan Frampton, Hagen Schwenzer, Gabriele Marino, Lee M. Butcher, Gabriele Pollara, Janos Kriston-Vizi, Cristina Venturini, Rachel Austin, Karina Ferreira de Castro, Robin Ketteler, Benjamin Chain, Richard A. Goldstein, Robin A. Weiss, Stephan Beck, and Ariberto Fassati

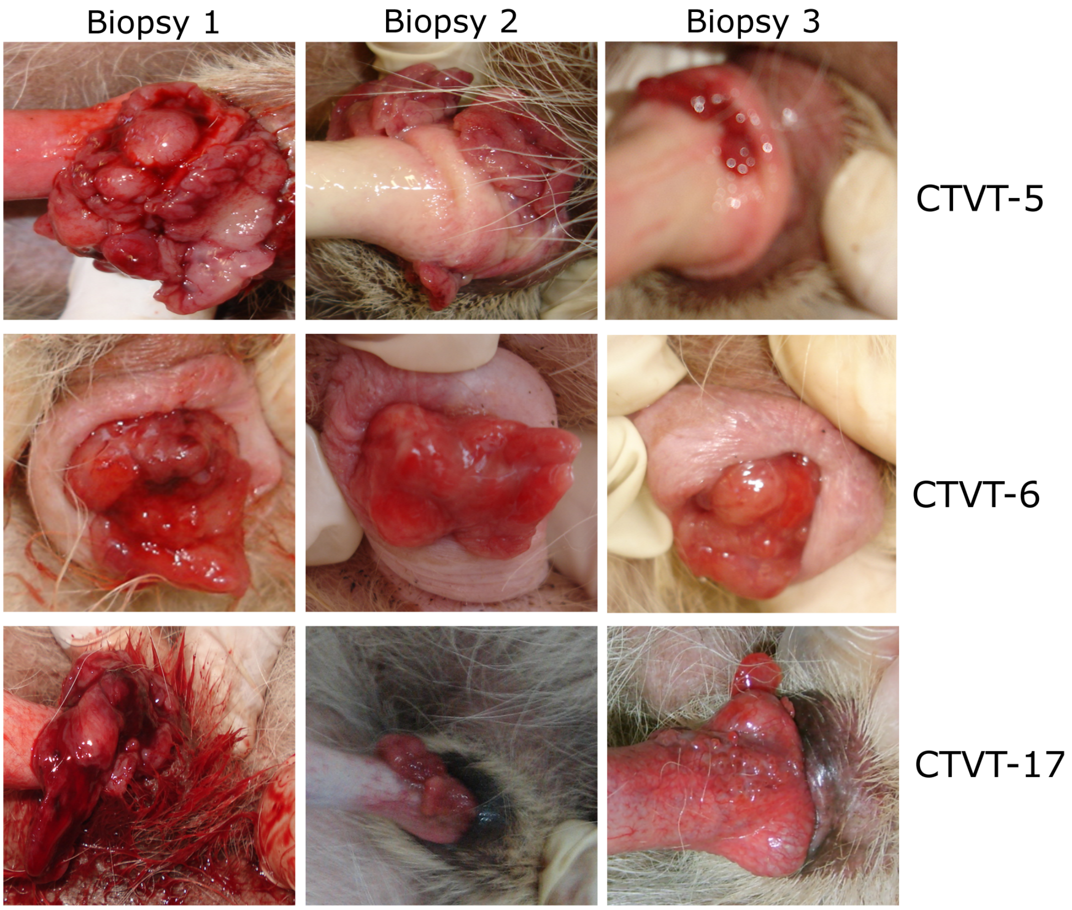

Figure S1 (related to Table 1).

Macroscopical appearance of CTVT cases diagnosed in Italy and used in this study for RNAseq and MeDIP-seq. CTVT-5 and CTVT-17 were diagnosed in male mixed breed dogs and CTVT-6 in a female mixed breed dog. Biopsies were collected at day 0, day 6 and day 14 after vincristine administration (CTVT-5 and CTVT-6), or at day 0, 22 and 48 after vincristine administration (CTVT-17). At the latest time point, CTVT-5 and CTVT-17 show overt clinical regression whereas CTVT-6 has clinical appearance of a tumor in stationary phase.

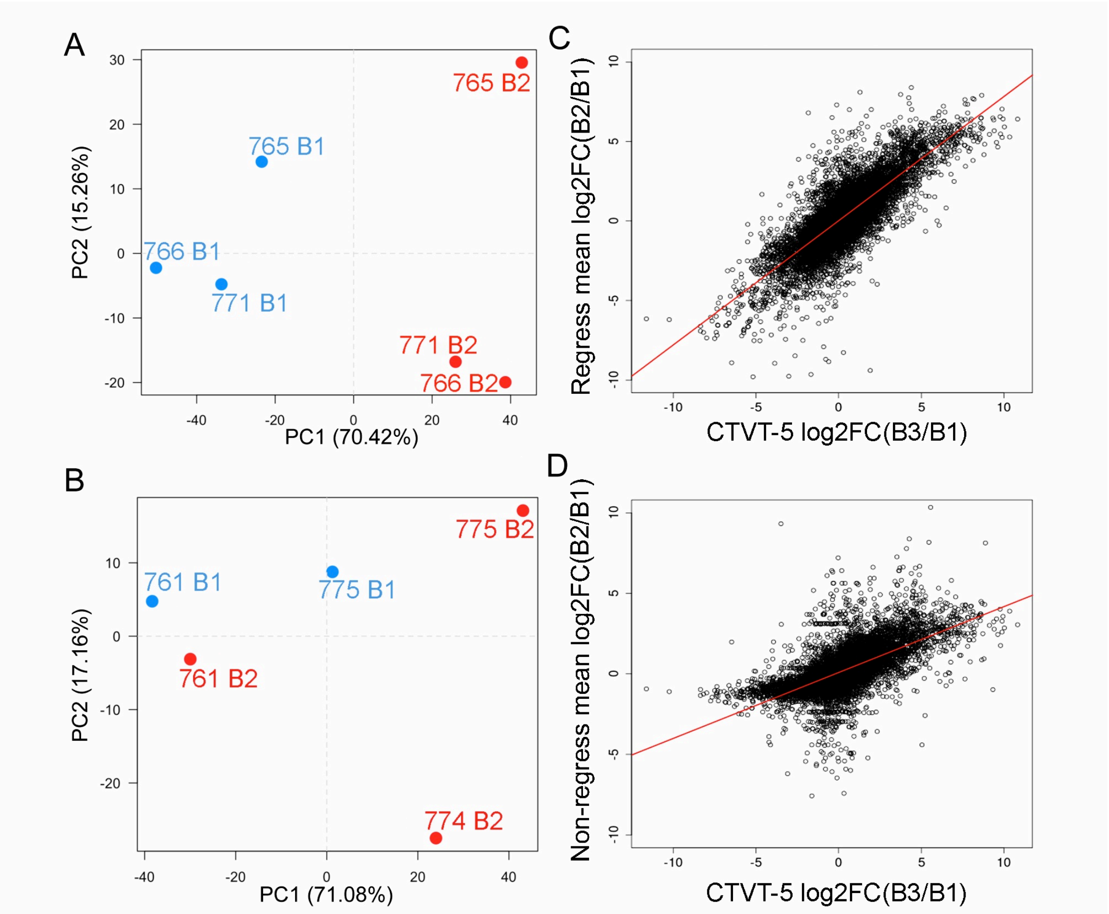

Figure S2 (related to Figure 2). Correlative analysis of regressive and non-regressive CTVTs.

(A, B) Principal component analysis (PCA) for the 7xx regressive (A) and non-regressive CTVTs (B). The figure shows the first two components of PCA with the percentage of variance associated with each axis. The first principal component (PC1) is expected to separate samples based on the different biological conditions, in blue biopsy 1 (B1) and in red biopsy 2 (B2). (C) correlation between the log2 fold-changes in gene expression in biopsy 3 (B3) relative to biopsy 1 (B1) of CTVT-5 (x-axis) and the mean log2 fold-changes in B2 relative to B1 in the regressive 7xx CTVTs. (D) correlation between the log2 fold-changes in gene expression in B3 relative to B1 of CTVT-5 (x-axis) and the mean log2 fold-changes for B2 relative to B1 in the non-regressive 7xx CTVTs. Pearson's correlation coefficient was calculated for both comparisons. Each dot represents a gene.

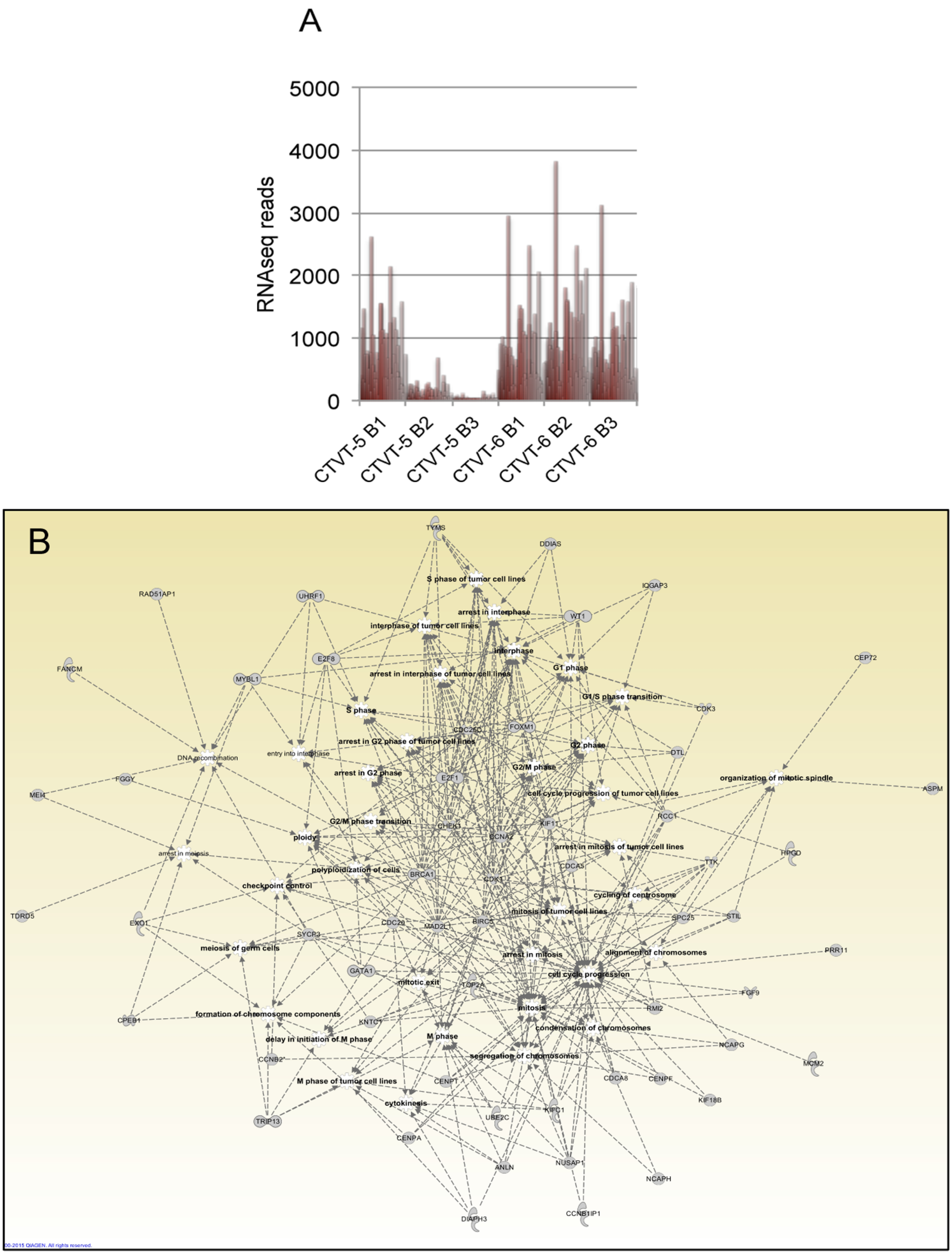

Figure S3 (related to Figure 6). Downregulation of cell cycle-related genes.

(A) Plot showing down-regulation of 60 cell cycle-related genes across the sequential biopsies of CTVT-5 and CTVT-6. B1: biopsy 1; B2: biopsy 2; B3: biopsy 3. The full list of

genes in provided in Table S7. (B) IPA diagram showing cell cycle gene networks of progressive downregulated genes in CTVT-5.

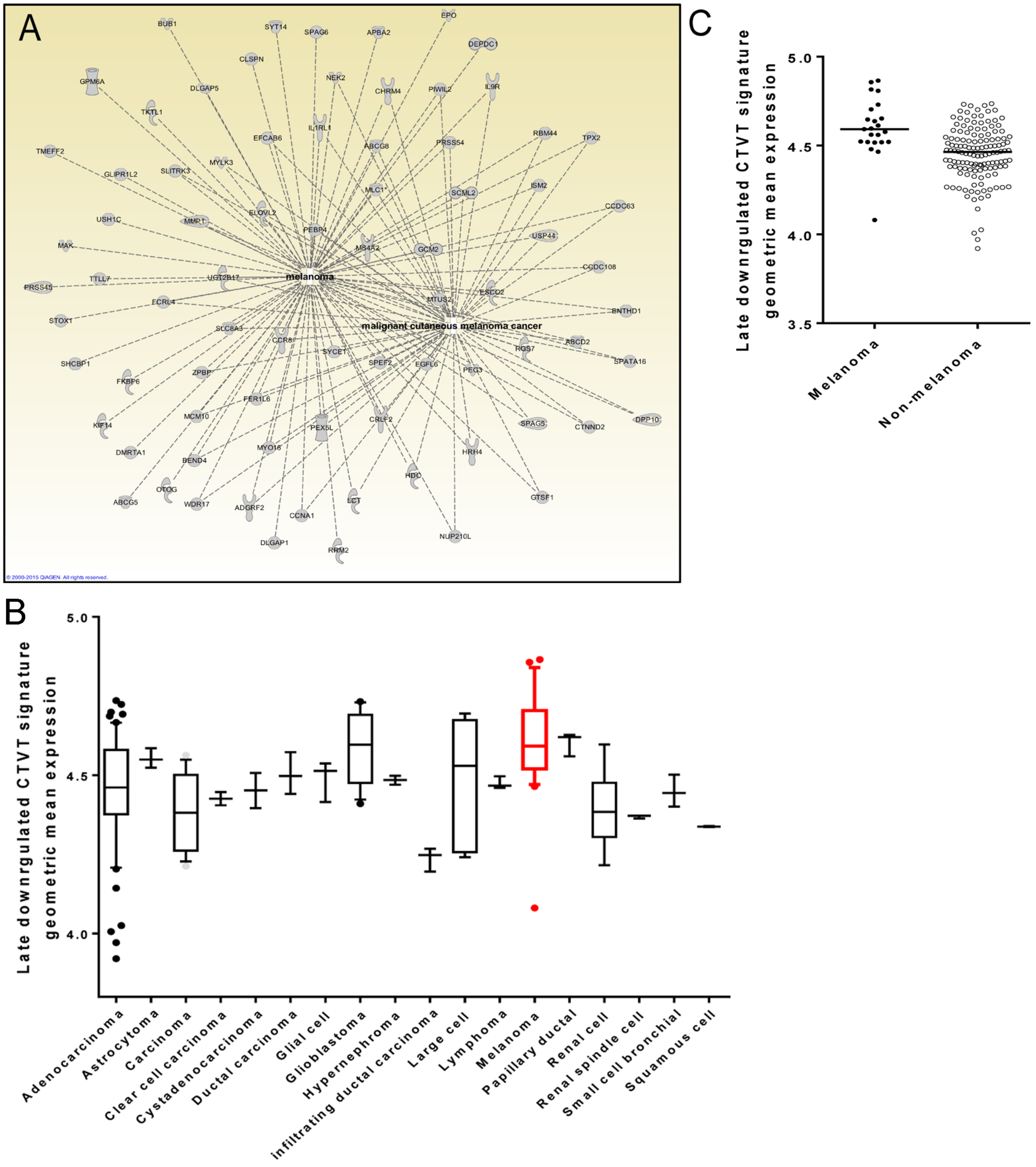

Figure S4 (related to Figure 6). Late down-regulated genes show a melanoma-like signature.

(A) IPA diagram showing melanoma/skin cancer gene networks of late downregulated genes in the third CTVT-5 biopsy. (B) Expression of late downregulated CTVT signature in NCI-60 cancer cell lines. Geometric mean expression of signature in multiple solid organ

cancer cell lines. Box represents the interquartile range and line represents median value. Whiskers are drawn to the 10th & 90th percentiles. (C) Comparison in geometric mean expression of late downregulated CTVT signature in melanoma cancer cells relative to all other solid organ cancer cells. Horizontal line represents median values, $p< 0.0001$ by Mann-Whitney test.

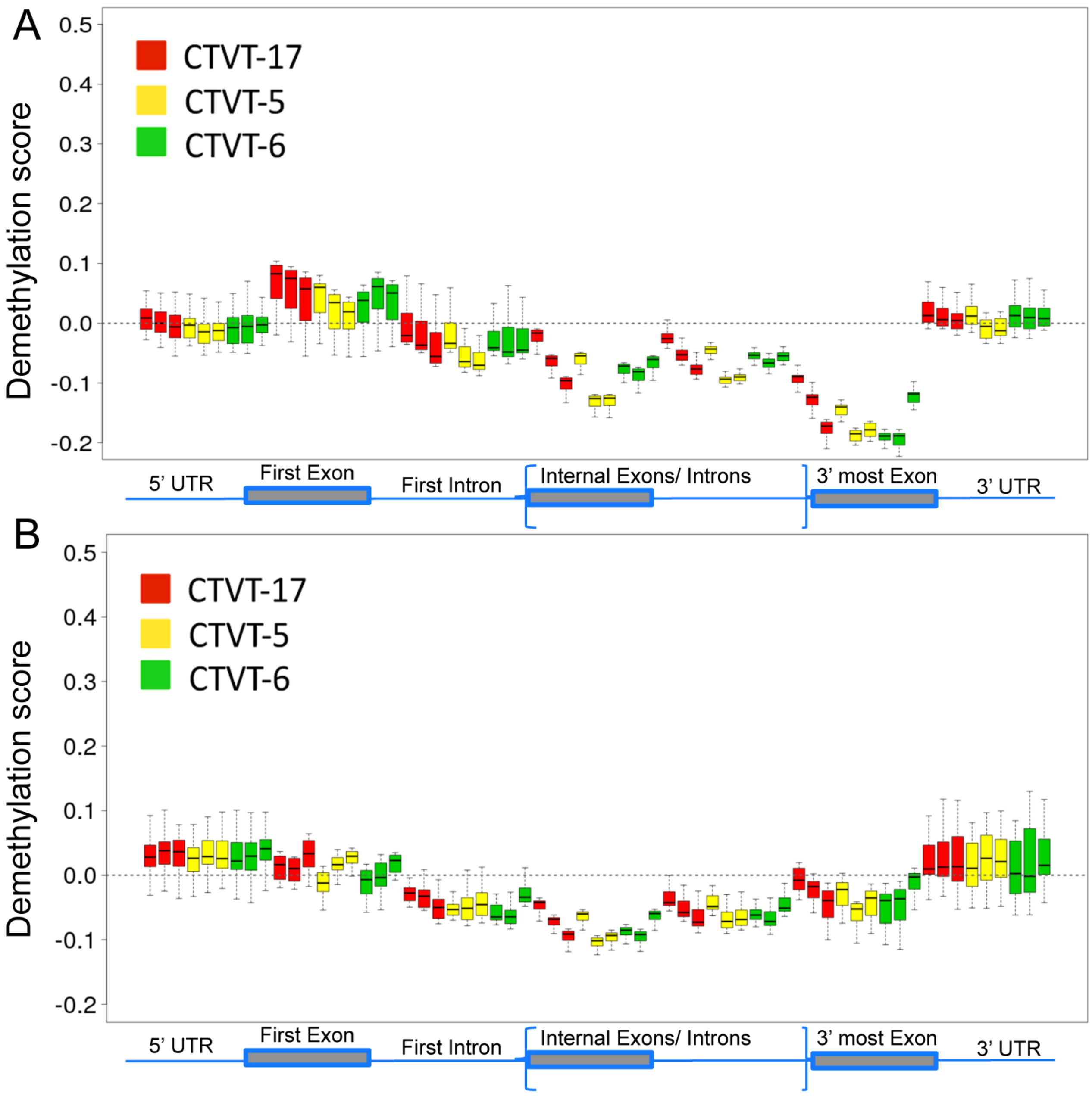

Figure S5 (related to Figure 7). Changes in gene expression correlate with specific changes in DNA methylation.

(A-B) De-methylation profiles of “early” (A) and “late” (B) downregulated genes across serial biopsies of CTVT-17 (red), CTVT-5 (yellow) and CTVT-6 (green). De-methylation scores were obtained for individual genes by quantifying demethylation levels within specific regions of genes (≤2 kb upstream of first exon; first exon; first intron; internal exons; internal introns; last exon; ≤2 kb downstream of last exon) and normalized by subtracting the corresponding demethylation values observed for non-expressed genes. Boxplots illustrate the variation within these values across each gene-list (boxes extend to the first and third

quartile, whiskers extend to 1.5x inter-quartile range and line represents median values). For each CTVT sample, boxplots are in order (from left to right): 1st, 2nd and 3rd biopsy.
